# Single-cell insights into cisplatin resistance mechanisms in bladder cancer tumor microenvironment

**DOI:** 10.1016/j.jbc.2026.111304

**Published:** 2026-02-20

**Authors:** Linfei Xu, Yongfeng Lin, Guanyun Shi, Liming Zhang, Xingzhang Lin, Tao Zhang

**Affiliations:** 1 Department of Urology, The Second Affiliated Hospital of Anhui Medical University, Hefei, China; 2 Department of Urology, Taizhou Municipal Hospital (Taizhou University Affiliated Municipal Hospital), School of Medicine, Taizhou University, Taizhou, Zhejiang, China

**Keywords:** bladder cancer, cisplatin resistance, single-cell RNA sequencing, cellular heterogeneity, cell–cell communication, metabolic reprogramming

## Abstract

This study integrates single-cell RNA sequencing with *in vitro* experimental validation to elucidate the molecular mechanisms underlying cisplatin resistance in bladder cancer (BC) and to characterize cellular heterogeneity within the tumor microenvironment. By analyzing integrated single-cell RNA sequencing datasets from cisplatin-sensitive and cisplatin-resistant BC samples, we identified key resistant cell subpopulations and resistance-associated signaling pathways. Notably, pronounced heterogeneity was observed among resistant epithelial cells and fibroblasts, accompanied by extensive metabolic reprogramming involving glycolysis, DNA damage repair, and drug metabolism pathways. Cell–cell communication analysis revealed intensified interactions between resistant cell subsets and immune cells or fibroblasts within the tumor microenvironment, with significant activation of macrophage migration inhibitory factor (MIF), thrombospondin, major histocompatibility complex-II, and fibronectin 1 signaling pathways. Developmental trajectory analysis further demonstrated the dynamic transition of fibroblasts from cisplatin-sensitive to -resistant states. Survival analyses across multiple cancer types confirmed the prognostic relevance of resistance-associated genes, including SPINK1, PHGR1, and APOD. Functional validation using a cisplatin-resistant BC cell line showed marked upregulation of SPINK1 following resistance induction. SPINK1 knockdown significantly reduced the cisplatin IC_50_ and suppressed MIF signaling. Moreover, resistant tumor cells enhanced macrophage tolerance to cisplatin *via* the MIF axis, an effect that was reversed by pharmacological MIF inhibition. Collectively, this integrated single-cell and experimental study reveals critical resistant cell subpopulations, metabolic reprogramming features, and intercellular communication networks driving cisplatin resistance in BC, highlighting potential molecular targets for therapeutic intervention and drug development.

Bladder cancer (BC) is one of the most common malignant tumors of the urinary system worldwide. Its incidence and mortality rates have been rising in recent years, posing a significant threat to public health (1). Although cisplatin-based chemotherapy is the gold standard for BC treatment and often achieves favorable initial clinical outcomes, the emergence of drug resistance leads to disease progression and recurrence in many patients. This significantly reduces overall survival and quality of life (2, 3, 4). Studies suggest that the development of resistance is a complex biological process involving various cellular and molecular mechanisms, such as the activation of drug efflux pumps, enhanced DNA damage repair (DDR), and intricate cell-to-cell interactions within the tumor microenvironment (TME) (5, 6). However, the molecular mechanisms by which cellular heterogeneity in TME drives the formation of drug resistance remain poorly understood. Addressing this question is critical for improving therapeutic efficacy and patient outcomes (7, 8).

In recent years, the TME, a complex ecological system, has garnered increasing attention for its role in the progression and drug resistance of BC. Epithelial cells, fibroblasts, and immune cells within the TME interact dynamically to influence tumor drug sensitivity through metabolic reprogramming, signaling pathway remodeling, and intercellular communication (9). Notably, phenomena such as enhanced glycolysis and the regulation of oxidative stress, key aspects of metabolic reprogramming, have been strongly linked to drug resistance (9, 10). However, existing studies often rely on population-level data, making it challenging to elucidate the unique functions and contributions of individual cells or specific subpopulations to drug resistance (11). Furthermore, traditional analytical methods exhibit significant limitations in capturing the dynamic changes within the TME and the intricate patterns of intercellular communication, thereby hindering a deeper understanding of the underlying mechanisms (12).

The development of single-cell omics technology has revolutionized the study of cellular heterogeneity and complex interactions within the TME (13). Unlike traditional population-level analyses, single-cell RNA sequencing (scRNA-Seq) enables precise classification of cellular subpopulations within tumors at single-cell resolution, uncovering heterogeneity in gene expression, metabolic activity, and signaling pathway dynamics (14). Moreover, tools such as Monocle allow for the tracking of developmental trajectories, providing insights into the dynamic evolution of tumor cells from drug sensitivity to resistance (15). By integrating metabolic analysis tools (*e*.*g*., scMetabolism) with cell–cell communication analysis platforms (*e*.*g*., CellChat and CellPhoneDB), researchers can comprehensively decode intercellular signaling networks and metabolic characteristics, offering new perspectives on the complex biological mechanisms underlying drug resistance (16). Thus, single-cell omics technology provides robust methodological support for addressing longstanding challenges in TME research.

Based on this, the present study employs a systematic approach that integrates single-cell omics with *in vitro* validation to elucidate the molecular underpinnings of cisplatin resistance in BC. To comprehensively decode the heterogeneity and resistance mechanisms inherent within the TME, we devised a multistage analytical framework. Initially, single-cell datasets from GSE135337 and GSE192575 were integrated to construct a unified cellular atlas, enabling a holistic characterization of the TME. Subsequently, our analysis centered on pivotal cell populations, specifically targeting epithelial and fibroblast subsets to pinpoint subpopulations unique to the cisplatin-resistant phenotype. We then conducted a multidimensional investigation—encompassing differential expression profiling, functional enrichment, intercellular communication (CellChat/CellPhoneDB), metabolic reprogramming (scMetabolism), and pseudotime trajectory inference (Monocle)—to unveil their biological attributes and potential mechanisms driving resistance. Finally, the identified core genes were independently validated using The Cancer Genome Atlas (TCGA) datasets and functional assays to ensure the robustness of our findings. Through this integrative analysis, we aim to clarify the functional characteristics of resistance-associated cells and confirm the prognostic significance of key resistance genes, thereby providing a crucial theoretical foundation for the development of novel targeted therapies. These insights hold promise for optimizing therapeutic strategies and enhancing long-term survival outcomes for patients with BC.

## Results

### Construction of the BC single-cell transcriptomic atlas and characterization of intercellular communication within the cisplatin-resistant microenvironment

**Figure 1:**
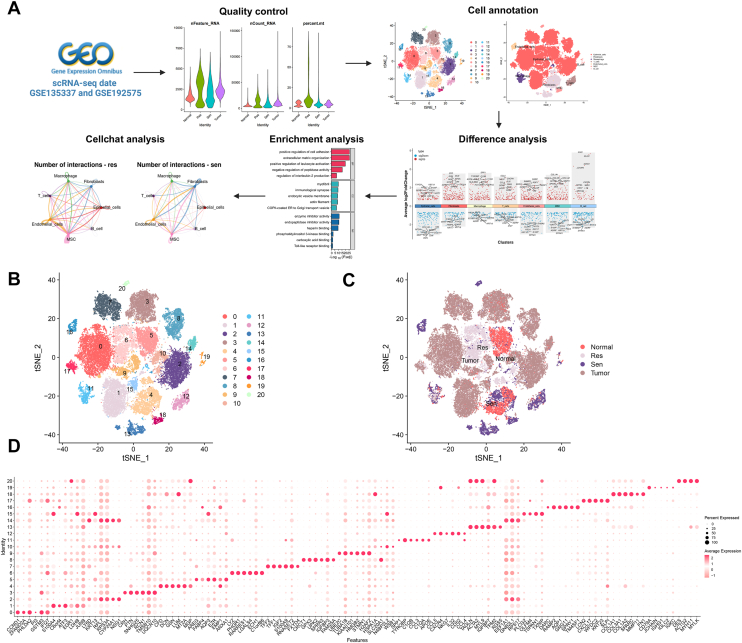
**Dimensionality reduction and cellular heterogeneity analysis of scRNA-Seq data**. *A*, workflow of scRNA-Seq data from tumor and matched normal tissues of BC patients in the Gene Expression Omnibus database. *B*, t-SNE dimensionality reduction of cells from cisplatin-sensitive (Sen, n = 1), cisplatin-resistant (Res, n = 1), tumor (Tumor, n = 6), and normal (Normal, n = 1) samples. *C*, distribution of cellular heterogeneity across cisplatin-sensitive (Sen, n = 1), cisplatin-resistant (Res, n = 1), tumor (Tumor, n = 6), and normal (Normal, n = 1) samples. *D*, expression trends of the top five DEGs in each cell cluster. This figure is based on scRNA-Seq data derived from GSE135337 (Tumor = 6, Normal = 1) and GSE192575 (Sen = 1, Res = 1). BC, bladder cancer; DEG, differentially expressed gene; scRNA-Seq, single-cell RNA sequencing; t-SNE, t-distributed stochastic neighbor embedding.

scRNA-Seq data from tumor tissues and matched normal tissues of BC patients, including cisplatin-sensitive and -resistant samples, were obtained from the Gene Expression Omnibus database (Fig. 1*A*). Quality control and filtering of the data were performed using the Seurat toolkit (Fig. S1). Cell quality was assessed based on metrics, such as nFeature_RNA, nCount_RNA, and percent.mt (Fig. S1*A*). The number of detected genes per cell (nFeature_RNA) ranged primarily from 1000 to 5000, whereas the total mRNA molecules per cell (nCount_RNA) were concentrated between 20,000 and 40,000. The proportion of mitochondrial genes (percent.mt) was generally below 10%. Cells were filtered using the criteria of 200 < nFeature_RNA < 5000 and percent.mt < 10%, retaining high-quality cells while removing low-quality ones. Scatterplots of nCount_RNA *versus* percent.mt and nCount_RNA *versus* nFeature_RNA were used to evaluate sequencing depth and quality. A weak correlation was observed between nCount_RNA and percent.mt (*r* = 0.07), whereas a strong positive correlation was identified between nCount_RNA and nFeature_RNA (*r* = 0.94), indicating that the number of expressed genes was highly correlated with the total number of mRNA molecules (Fig. S1*B*). This strong correlation further validated the high sequencing quality of the retained cells, enabling them to proceed to downstream analyses. Following Seurat integration and Harmony-based batch correction, all samples (Normal, Tumor, Sen, and Res) were jointly projected into a unified low-dimensional space for subsequent clustering and differential analyses.

The top 2000 highly variable genes were selected based on gene expression variance (Fig. S1*C*) and subsequently used for downstream analyses. Cell cycle stages were annotated using the CellCycleScoring method, illustrating the distribution of scores for cells in the S and G2M phases (Fig. S1*D*). Principal component analysis was performed on the highly variable genes to reduce dimensionality, and a heatmap of the top genes associated with the first six principal components was generated (Fig. S1*E*). To evaluate batch effects, the distribution of cells along principal component_1 and principal component_2 was analyzed, revealing some batch-specific variations among samples (Fig. S1*F*). To address this, the Harmony algorithm was applied for batch effect correction, resulting in improved clustering consistency in the corrected data (Fig. S1*G*).

The t-distributed stochastic neighbor embedding (t-SNE) algorithm was applied to perform nonlinear dimensionality reduction on cells from BC and normal tissues, revealing their distribution patterns (Fig. 1*B*). The t-SNE clustering identified 21 distinct cell clusters, each representing different cellular subpopulations within the tumor and its microenvironment. By grouping cells from various samples, significant cellular heterogeneity was observed among cisplatin-sensitive (Sen), cisplatin-resistant (Res), and tumor (Tumor) BC samples (Fig. 1*C*). Clustering across different resolutions was visualized using the clustree package (Fig. S2*A*), and a resolution of 0.3 was selected, resulting in 21 clusters. These clusters were annotated based on the expression profiles of their respective cellular subpopulations. Analysis of the top five differentially expressed genes (DEGs) in each cluster revealed distinct expression trends across the clusters (Figs. 1*D*, S2*B*), providing insights into the potential molecular mechanisms underlying cellular heterogeneity within the TME.

Marker gene expression profiles of each cell cluster, identified through clustering, were annotated using the SingleR tool for scRNA-Seq data. This analysis revealed distinct cell types, including epithelial cells, fibroblasts, macrophages, T cells, endothelial cells, mesenchymal stem cells, and B cells (Fig. S3*A*). Using the t-SNE algorithm, these cell populations were visualized, demonstrating clear distribution patterns in the dimensionality-reduced space (Fig. S3*B*). Epithelial cells occupied a prominent position in the t-SNE space, with a noticeably higher abundance in the Res group compared with the Sen group (Fig. S3*C*). A statistical analysis of cell counts across samples further confirmed that epithelial cells were significantly more abundant than other cell types, particularly in the Tumor group (BC1–BC6) and the Res group (Fig. S3*D*). Comparative analysis of cell population distributions across groups (Fig. S3*E*) showed that the proportion of epithelial cells was markedly increased in tumor tissues and drug-resistant samples, whereas fibroblasts were more prevalent in normal and drug-sensitive samples. These findings suggest a potential role for epithelial cells and fibroblasts in BC drug resistance. Visualization of marker gene expression across cell types (Fig. S3, *F*–*G*) revealed distinct patterns for genes, such as KRT19, CFD, SPP1, CCL5, DARC, COL1A1, and IGKC. These differential expression patterns further indicate that these genes may play critical roles in regulating tumor cell heterogeneity and resistance to therapy.

To investigate the cooperative mechanisms by which these heterogeneous cells drive drug resistance within the TME, we performed a comprehensive analysis of intercellular differential gene expression and communication networks.

**Figure 2:**
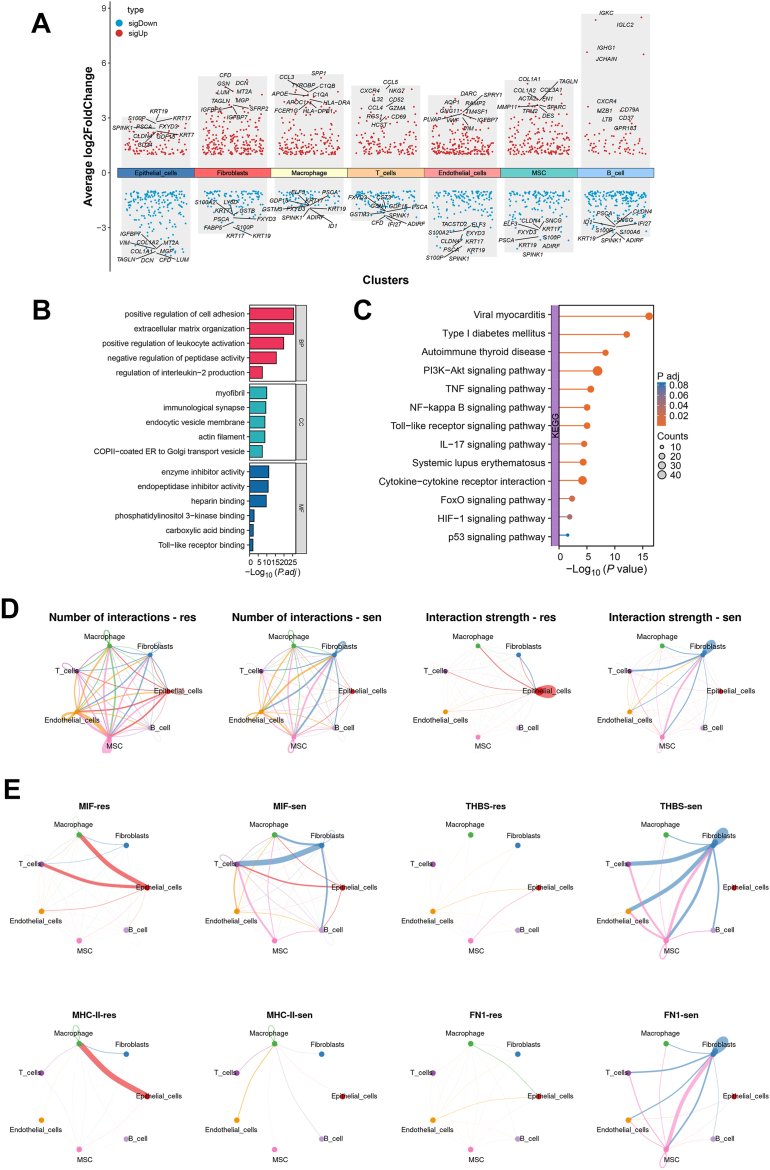
**scRNA-Seq reveals DEGs and signaling pathways associated with cisplatin resistance in BC**. *A*, volcano plot illustrating DEGs associated with cisplatin resistance in BC. *B*, Gene Ontology (GO) functional enrichment analysis highlighting the functional pathways of DEGs. *C*, KEGG pathway enrichment analysis showing metabolic pathways of DEGs. *D*, CellChat analysis comparing the quantity and strength of intercellular communication between the resistant (Res) and sensitive (Sen) groups. *E*, activity analysis of specific signaling pathways across different cell types. Groups include the cisplatin-sensitive group (Sen, n = 1), cisplatin-resistant group (Res, n = 1), tumor group (Tumor, n = 6), and normal group (Normal, n = 1). All analyses in this figure are based on scRNA-Seq data from GSE135337 (Tumor: GSM4006644, GSM4006645, GSM4006646, GSM4006647, GSM4006648, and GSM4751267; Normal: GSM5329919) and GSE192575 (Sen: GSM5751919; Res: GSM5751918). BC, bladder cancer; DEG, differentially expressed gene; KEGG, Kyoto Encyclopedia of Genes and Genomes; scRNA-Seq, single-cell RNA sequencing.

Using scRNA-Seq, differential analysis was performed on each cell cluster across all samples, identifying key genes associated with tumor progression, inflammatory responses, and extracellular matrix remodeling (Fig. 2*A*). DEG analysis revealed significant upregulation and downregulation of genes in epithelial cells, fibroblasts, macrophages, T cells, endothelial cells, mesenchymal stem cells, and B cells (avg_log2 fold change [FC] = 1, *p*_val_adj = 0.05). Notably, genes upregulated in epithelial cells (*e*.*g*., FXYD3, KRT19, S100P, SPINK1, CLDN4, KRT7, CD24, GDF15) were implicated in inflammatory responses, tumor proliferation, and invasion processes (17, 18, 19). Further Gene Ontology enrichment analysis of these DEGs (Fig. 2*B*) revealed significant associations with processes, such as cell adhesion, extracellular matrix organization, inflammatory responses, and immune responses, which are closely linked to the invasiveness and drug resistance of BC cells (20, 21). In addition, functions related to the endoplasmic reticulum and cytoskeleton were enriched, suggesting these genes may play essential roles in regulating cell migration, proliferation, and survival (22). Kyoto Encyclopedia of Genes and Genomes (KEGG) pathway enrichment analysis highlighted the involvement of these genes in signaling pathways associated with drug resistance and tumor metabolic reprogramming (Fig. 2*C*). Pathways such as PI3K-Akt, TNF, NF-kappa B, Toll-like receptor, HIF-1, FoxO, and p53 were significantly enriched. These pathways are critical in regulating tumor cell proliferation, survival, metabolism, apoptosis, and resistance to chemotherapy (23, 24). In particular, activation of the HIF-1 and PI3K–Akt pathways suggests that tumor cells may adapt to hypoxic conditions through metabolic reprogramming, thereby enhancing resistance to chemotherapeutic agents (25, 26).

To investigate the communication patterns between different cell types within the TME, analysis was conducted using CellChat. The results revealed significant differences between the Res and Sen groups (Fig. 2*D*). Specifically, the number and intensity of intercellular communications were markedly increased in the resistant group, with the interactions between epithelial cells and fibroblasts being particularly active. This suggests that these cell types may play critical roles in the development of resistance. In contrast, the communication pathways in the sensitive group showed relatively lower activity. Further analysis of specific signaling pathways (Fig. 2*E*) indicated that pathways, such as macrophage migration inhibitory factor (MIF), thrombospondin (THBS), major histocompatibility complex II (MHC-II), and fibronectin 1 (FN1), were significantly more active in the resistant group, especially in interactions involving epithelial cells and other cell types. The MIF pathway is closely associated with tumor immune evasion and inflammatory responses (27), whereas the THBS pathway is linked to extracellular matrix remodeling (28). The MHC-II pathway plays a role in immune regulation (29), and the FN1 pathway is involved in cell adhesion and migration (30). The abnormal activation of these pathways may represent key drivers of cisplatin resistance in BC.

Using the CellPhoneDB database, detailed interaction analyses were performed for the Res and Sen groups (Fig. S4*A*). The results showed that the MDK–NCL and LAMC1–ITGA6 signaling pathways were more active in the resistant group. These pathways are critically involved in tumor progression, cell proliferation, and antiapoptotic processes (31, 32), suggesting their potential role in the development of cisplatin resistance in BC cells. Notably, interactions between MHC class I molecules (*e*.*g*., HLA-E, HLA-F) and their receptors (CD94, NKG2A), which are associated with immune evasion, were significantly enhanced in the resistant group, indicating that tumor cells may leverage immune evasion mechanisms to strengthen resistance (33). These ligand–receptor (L–R) pairs likely promote cell adhesion and migration, contributing to the formation of a resistance advantage. Relative information flow analysis further revealed (Fig. S4*B*) that signaling pathways, such as MHC-I, GDF, and GRN, were significantly upregulated in the resistant group, with a particularly strong trend in the MHC-I pathway. This suggests that resistant cells may counteract cisplatin treatment through mechanisms involving antigen presentation and immune evasion (34).

Signal transduction analysis further validated these findings (Fig. S5, *A*–*B*). In the resistant group (Res), both incoming and outgoing signaling pathways were significantly enhanced, particularly those involving communication between fibroblasts, macrophages, and epithelial cells. Immunosuppressive signaling pathways (*e*.*g*., MHC-I) (35), proangiogenic pathways (*e*.*g*., vascular endothelial growth factor) (36), and pathways associated with extracellular matrix remodeling (*e*.*g*., COLLAGEN, FN1) (37) exhibited higher signaling activity in the Res group. The amplification of these pathways suggests that CellChat in the TME plays a critical role in the development of cisplatin resistance, potentially by regulating tumor cell survival, migration, and immune evasion.

These results highlight the complex communication networks and L–R interaction mechanisms between different cell types in the TME during the formation of cisplatin resistance in BC. Epithelial cells in BC engage in crosstalk with immune cells and fibroblasts, activating multiple signaling pathways associated with drug resistance and immunosuppression, which may play a pivotal role in cisplatin resistance. Unraveling these communication mechanisms provides potential molecular targets for the treatment of BC.

### Identification of heterogeneity in cisplatin-resistant epithelial subpopulations and pseudotime evolutionary trajectories

**Figure 3:**
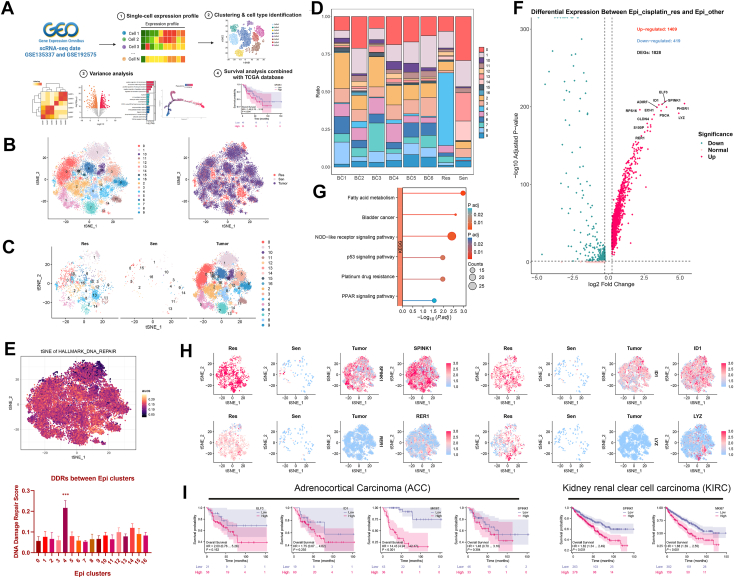
**Subpopulation analysis of BC epithelial cells reveals heterogeneity and DEGs associated with cisplatin resistance**. *A*, workflow diagram of the Epi_cisplatin_res analysis in BC. *B*, t-SNE dimensionality reduction illustrating the clustering of epithelial cells in cisplatin-sensitive (Sen, n = 1), cisplatin-resistant (Res, n = 1), and tumor (Tumor, n = 6) samples. *C*, comparative distribution of epithelial cells across different groups. *D*, proportion of each epithelial cell subpopulation across individual samples. *E*, AUC quantification showing DDRs in epithelial cell subpopulations. *F*, volcano plot displaying DEGs between Epi_cisplatin_res and Epi_other. *G*, KEGG pathway enrichment analysis highlighting key pathways involving DEGs. *H*, expression patterns of DEGs in epithelial cells from the Res, Sen, and Tumor groups. *I*, survival analysis based on the TCGA database showing the association of cisplatin resistance–related genes with overall survival in ACC (n = 79) and KIRC (n = 613). KEGG, Kyoto Encyclopedia of Genes and Genomes. ACC, adrenocortical carcinoma; AUC, area under the curve; BC, bladder cancer; DEG, differentially expressed gene; DDR, DNA damage repair; Epi_cisplatin_res, epithelial cisplatin–resistant subpopulation; Epi_other, epithelial nonresistant subpopulation; KIRC, kidney renal clear cell carcinoma; TCGA, The Cancer Genome Atlas; t-SNE, t-distributed stochastic neighbor embedding.

Unsupervised clustering analysis of scRNA-Seq data from BC epithelial cells identified multiple subpopulations associated with cisplatin resistance (Fig. 3*A*). Based on single-cell clustering results (Fig. 3, *B* and *C*), epithelial cells exhibited significant heterogeneity between the cisplatin-sensitive group (Sen) and the resistant group (Res), with notable differences concentrated in cluster 4. This suggests that cluster 4 in the Res group may play a critical role in cisplatin resistance. Notably, the proportion of epithelial cells within cluster 4 was higher in the Res group, further indicating the potential involvement of these subpopulations in mediating cisplatin resistance (Fig. 3*D*).

Further area under the curve (AUC) quantification analysis revealed that cluster 4 in the Res group exhibited significantly higher DDR Score (DDRs), suggesting that this cell subset may constitute the primary population of resistant cells (Fig. 3*E*). These clusters were defined as the resistant subgroup (Epi_cisplatin_res), whereas other clusters were classified as the nonresistant subgroup (Epi_other). In the t-SNE dimensionality reduction space, Epi_cisplatin_res cells were predominantly distributed within the Res group, with their proportion notably higher in the Res group compared with the Sen group, indicating their critical role in cisplatin resistance (Fig. S6, *A*–*B*). Further analysis of the top five marker genes in Epi_cisplatin_res and Epi_other cell populations (Fig. S6*C*) revealed markedly higher expression of marker genes (LYZ, PHGR1, APOD, RARRES1, and KLK2) in the Epi_cisplatin_res subgroup. Notably, APOD has been implicated in tumor growth and metastasis, and its elevated expression is associated with resistance to chemotherapy drugs like cisplatin (38), suggesting its pivotal role in cisplatin resistance in BC cells.

The volcano plot analysis of DEGs (Fig. 3*F*) further highlights the differences between the Epi_cisplatin_res and Epi_other groups. A total of 1559 upregulated and 419 downregulated genes were identified. Notably, genes closely associated with chemoresistance, such as SPINK1, MKI67, and RER1, were significantly overexpressed in the resistant subgroup. These genes likely contribute to chemoresistance by promoting cell proliferation and inhibiting apoptosis (39, 40). KEGG enrichment analysis (Fig. 3*G*) revealed that the DEGs were predominantly involved in pathways, such as fatty acid metabolism, nucleotide-binding oligomerization domain–like receptor signaling, the p53 signaling pathway, platinum drug resistance, and the peroxisome proliferator–activated receptor signaling pathway. In particular, the activation of fatty acid metabolism, platinum drug resistance, and the p53 and peroxisome proliferator–activated receptor signaling pathways may be closely linked to metabolic reprogramming in tumor cells and the development of chemoresistance (41, 42).

The differential genes were mapped to the expression profiles of epithelial cells to further validate their expression patterns across different groups (Fig. 3*H*). The results revealed significant differences in the expression of key differential genes (SPINK1, RER1, ID1, and LYZ) among the Res, Sen, and Tumor groups. In the Res group, SPINK1, RER1, and ID1 exhibited significantly higher expression levels compared with the Sen group. Notably, SPINK1 showed elevated expression in the Epi_cisplatin_res subcluster, suggesting its potential role in regulating cell proliferation and survival, thereby contributing to the mechanisms of BC resistance (17). Similarly, the high expression of ID1 and RER1 indicates their critical roles in drug resistance (43). In the Tumor group, LYZ displayed markedly increased expression, implying its important immunoregulatory function within the TME (44). Furthermore, the elevated expression of ID1 and SPINK1 in the Tumor group provides additional support for their potential roles as resistance-associated genes.

These findings suggest that the Epi_cisplatin_res subpopulation is strongly associated with cisplatin resistance. The gene expression profiles not only reveal the heterogeneity within the epithelial cell subpopulations but also identify potential resistance-related targets, providing a foundation for in-depth studies on the mechanisms of cisplatin resistance in BC. Survival analysis of cisplatin resistance–related genes using the TCGA database indicates that these genes are associated with poor prognosis across various cancers (Fig. 3*I*). In adrenocortical carcinoma (ACC), patients with high expression of EIF3I, ID1, and MKI67 exhibit significantly reduced overall survival, suggesting that these genes may serve as biomarkers of poor prognosis in ACC. Similarly, in kidney renal clear cell carcinoma (KIRC), high expression of SPINK1 and MKI67 correlates with lower overall survival, further highlighting their potential roles in tumor progression and cisplatin resistance.

In light of the unique resistance profile of the Epi_cisplatin_res subpopulation, we utilized pseudotime analysis to trace its dynamic evolutionary trajectory from cisplatin sensitivity to resistance.

Pseudotime analysis of BC epithelial cells revealed significant differences in differentiation trajectories between Epi_cisplatin_res and Epi_other cells (Fig. S7, *A*–*C*). The results showed that Epi_cisplatin_res cells progressed along the pseudotime trajectory to a more advanced differentiation state, indicating that these cells undergo specific differentiation stages during the development of cisplatin resistance. In contrast, Epi_other cells predominantly occupied early to intermediate differentiation states, suggesting a higher sensitivity to cisplatin treatment. These trajectory differences were further validated by state distribution analysis, where Epi_cisplatin_res cells were predominantly localized in state 7 (Fig. S7*D*), suggesting that these cells may have completed differentiation and acquired high resistance characteristics.

Analysis of changes in key transcription factor expression (Fig. S7*E*) revealed a progressive increase in the expression levels of transcription factors associated with cell proliferation, migration, and drug resistance (CD44, CD74, CFH, CYP24A1, PLAUR, PSMC4, RALBP1, and SNAI2) during the pseudotime progression of Epi_cisplatin_res cells. These expression trends highlight the distinct differentiation characteristics of Epi_cisplatin_res cells during the development of drug resistance. Notably, CD44 and SNAI2, identified as key regulatory factors related to cell proliferation, migration, and resistance (45), showed significant upregulation at later differentiation stages of Epi_cisplatin_res cells. This suggests that these genes may contribute to cisplatin resistance in BC cells by influencing cell adhesion and epithelial–mesenchymal transition. In addition, the upregulation of genes, such as CYP24A1 and PLAUR, further underscores the roles of metabolic regulation and extracellular matrix remodeling in the development of drug resistance (46).

The heatmap analysis (Fig. S7*F*) further revealed the dynamic changes in DEGs during the pseudotime progression. In the prebranch phase of the pseudotime trajectory, several genes, including RPL6, EIF4B, GSTP1, and SNAI2, were significantly upregulated. Both RPL6 and EIF4B are associated with protein synthesis, suggesting that cells in this phase may exhibit a heightened proliferative state (47). GSTP1, a member of the glutathione-*S*-transferase family, is linked to oxidative stress resistance induced by cisplatin (48). SNAI2, typically associated with epithelial–mesenchymal transition, indicates that tumor cells may begin acquiring invasive and migratory capabilities at this stage (49). In the cell fate 1 branch, the expression of CXCL2, PLPP1, and PKM was elevated. CXCL2 may promote tumor cell migration by modulating immune responses within the TME (50). PKM, a key enzyme in glycolysis, suggests a shift toward glycolysis as a primary energy source for these cells (51). PLPP1 may regulate phosphorylation processes, contributing to cellular stress responses and metabolic regulation (52). In the cell fate 2 branch, genes such as SIRT2 and LYZ, which are associated with cell migration, invasion, and cisplatin resistance, were upregulated. SIRT2 plays a role in cell cycle regulation, DNA repair, and stress responses, indicating its involvement in mechanisms conferring resistance to cisplatin-induced DNA damage and oxidative stress (53). LYZ, related to inflammation and immune responses, suggests that cells may evade host immune surveillance by altering the immune microenvironment at this stage (44). These genes are implicated in tumor cell migration, invasion, stress responses, DNA repair, and immune evasion, facilitating resistance to cisplatin-induced cellular damage and apoptosis. These findings provide new insights into the development of cisplatin resistance in BC and highlight potential therapeutic targets.

### Molecular characterization, metabolic reprogramming, and developmental dynamics of cisplatin-resistant fibroblast subpopulations

**Figure 4:**
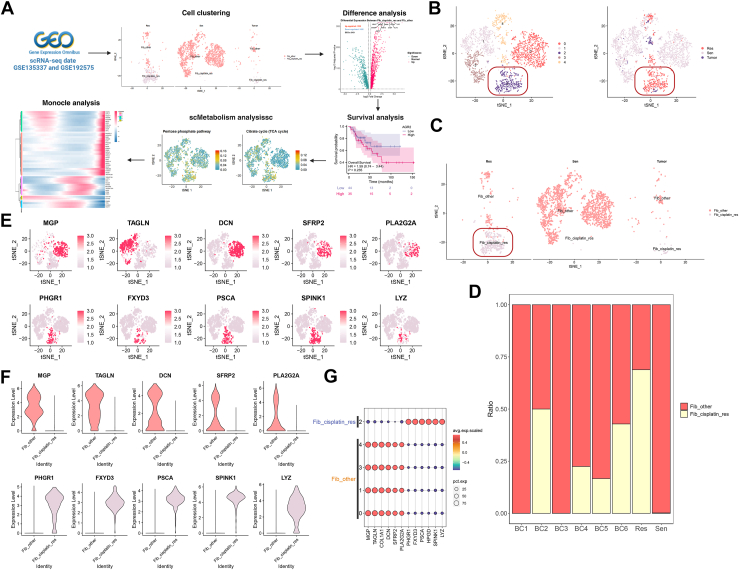
**scRNA-Seq analysis of BC fibroblasts reveals heterogeneity and marker genes associated with cisplatin resistance**. *A*, workflow for analyzing Fib_cisplatin_res in BC. *B*, distribution of fibroblasts from the Res and Sen groups in the t-SNE dimensionality reduction space. *C*, t-SNE visualization of the distribution of Fib_cisplatin_res and Fib_other subpopulations across different groups. *D*, proportional analysis of Fib_cisplatin_res and Fib_other subpopulations in different samples. *E*, *feature plot* showing the expression levels of the top 5 marker genes in Fib_cisplatin_res and Fib_other subpopulations. *F*, *box plot* illustrating the expression patterns of the top 5 marker genes in different subpopulations. *G*, *dot plot* depicting the expression characteristics of key marker genes in Fib_cisplatin_res and Fib_other. BC, bladder sancer; Fib_cisplatin_res, fibroblast cisplatin–resistant subpopulation; Fib_other, fibroblast nonresistant subpopulation; Res, cisplatin-resistant group; scRNA-Seq, single-cell RNA sequencing; Sen, cisplatin-sensitive group; t-SNE, t-distributed stochastic neighbor embedding.

Unsupervised clustering analysis identified distinct fibroblast subpopulations in BC (Fig. 4*A*). Notably, in the Sen and Res groups, clustering results revealed significant fibroblast heterogeneity (Fig. 4*B*). The primary differences between the Res and Sen groups were concentrated in cluster 2, which was subsequently defined as the Fib_cisplatin_res, whereas other subpopulations were categorized as Fib_other. In the t-SNE dimensionality reduction plot, Fib_cisplatin_res cells were predominantly enriched in the Res group, whereas their presence was markedly reduced in the Sen group, further suggesting a strong association between this subpopulation and cisplatin resistance (Fig. 4*C*). Cellular proportions across samples (Fig. 4*D*) showed a significantly higher proportion of Fib_cisplatin_res cells in the Res group compared with the Sen group. This finding indicates that the Fib_cisplatin_res subpopulation may play a critical role in the development of cisplatin resistance in BC, potentially providing a supportive microenvironment for tumor cells to acquire resistance. Further analysis of the top five marker genes expressed in Fib_cisplatin_res and Fib_other subpopulations revealed that PHGR1, FXYD3, PSCA, SPINK1, and LYZ were significantly upregulated in Fib_cisplatin_res but showed lower expression in Fib_other (Fig. 4, *E*–*G*). PHGR1 is associated with cell proliferation and migration and has been implicated in tumor progression (54). PSCA is highly expressed in various cancers and is linked to tumor growth and metastasis (55). SPINK1 contributes to cisplatin resistance mechanisms in BC by regulating cell proliferation and survival (17). LYZ is involved in modulating inflammatory responses and immune activity under different conditions (44). FXYD3 maintains cellular electrolyte balance during stress responses and enhances tolerance to cisplatin-induced stress (56). The elevated expression of these genes in the Fib_cisplatin_res subpopulation is closely linked to tumor cell proliferation, migration, and resistance to cisplatin, suggesting their pivotal role in the development of cisplatin resistance in BC. The Fib_cisplatin_res subpopulation may promote tumor cell survival and resistance to cisplatin through the upregulation of these key genes.

**Figure 5:**
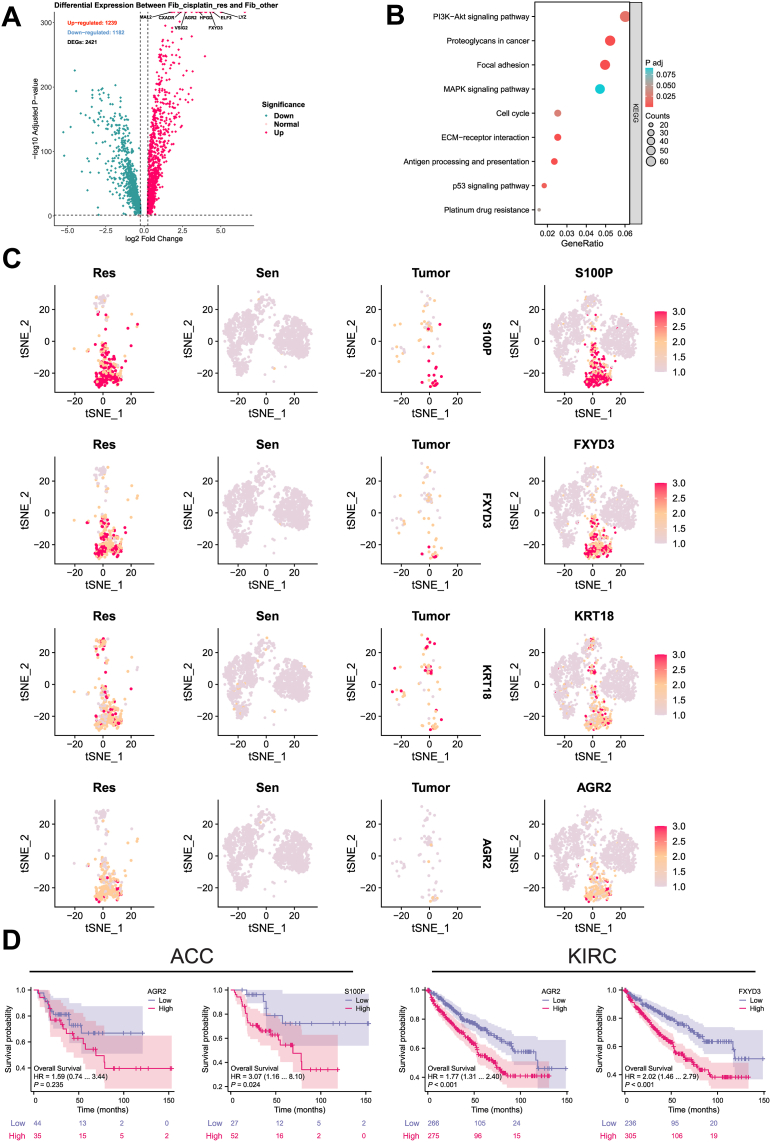
**Differential gene analysis and prognostic relevance of Fib_cisplatin_res in BC**. *A*, volcano plot illustrating the differential genes between Fib_cisplatin_res and Fib_other subpopulations. *B*, KEGG pathway enrichment analysis highlighting key signaling pathways associated with differential gene. *C*, expression patterns of differential genes in Res, Sen, and Tumor groups of fibroblasts. *D*, survival analysis showing the prognostic significance of cisplatin resistance–related genes in ACC (n = 79) and KIRC (n = 613). ACC, adrenocortical carcinoma; BC, blood cancer; Fib_cisplatin_res, fibroblast cisplatin–resistant subpopulation; Fib_other, fibroblast nonresistant subpopulation; KEGG, Kyoto Encyclopedia of Genes and Genomes; KIRC, kidney renal clear cell carcinoma; Res, cisplatin-resistant group; Sen, cisplatin-sensitive group; Tumor, tumor group.

To clarify the specific molecular mechanisms and pathways driving resistance in this subpopulation, we performed differential gene expression and functional enrichment analyses. Volcano plot analysis of the DEGs between the Fib_cisplatin_res and Fib_other subgroups identified several key genes associated with cisplatin resistance in BC (Fig. 5*A*). These genes were significantly upregulated in the resistant subgroup, suggesting their crucial roles in resistance development. KEGG enrichment analysis (Fig. 5*B*) revealed that these DEGs are primarily involved in immune cell adhesion and migration, promotion of glycolysis, DDR, drug efflux, and apoptosis inhibition. Pathways such as focal adhesion, PI3K–Akt signaling, mitogen-associated protein kinase signaling, and platinum drug resistance were strongly associated with cisplatin resistance (57, 58), highlighting their potential roles in the resistance mechanisms of BC cells. Mapping these DEGs onto single-cell data (Fig. 5*C*) revealed that genes such as S100P, FXYD3, KRT18, and AGR2 were highly expressed in the Fib_cisplatin_res subgroup, further confirming its pivotal role in cisplatin resistance. Notably, S100P has been implicated in tumor growth and metastasis and is associated with drug resistance (59). KRT18 is linked to cell migration and invasion (60). FXYD3 plays a role in maintaining cellular electrolyte balance under stress, enhancing tolerance to cisplatin-induced stress responses (56). AGR2 is involved in resistance across various tumors (61). Survival analysis using the TCGA database demonstrated that cisplatin resistance–related genes are significantly associated with poor prognosis in multiple cancer types (Fig. 5*D*). In ACC, high expression of AGR2 and S100P was correlated with significantly reduced overall survival, suggesting their roles as prognostic indicators linked to tumor progression and resistance. Similarly, in KIRC, elevated expression of AGR2 and FXYD3 was negatively correlated with overall survival, further supporting their involvement in tumor progression and chemotherapy resistance. These findings indicate that the DEGs in the Fib_cisplatin_res subgroup may serve not only as potential drivers of cisplatin resistance but also as prognostic markers closely associated with tumor outcomes in multiple cancers. Collectively, these genes hold promise as clinical biomarkers and therapeutic targets for BC and other malignancies.

In addition to transcriptomic features, metabolic reprogramming is a distinct characteristic of drug-resistant cells. Consequently, we combined metabolic pathway enrichment with pseudotime analysis to unravel the metabolic evolution of the cisplatin-resistant fibroblast subpopulation.

**Figure 6:**
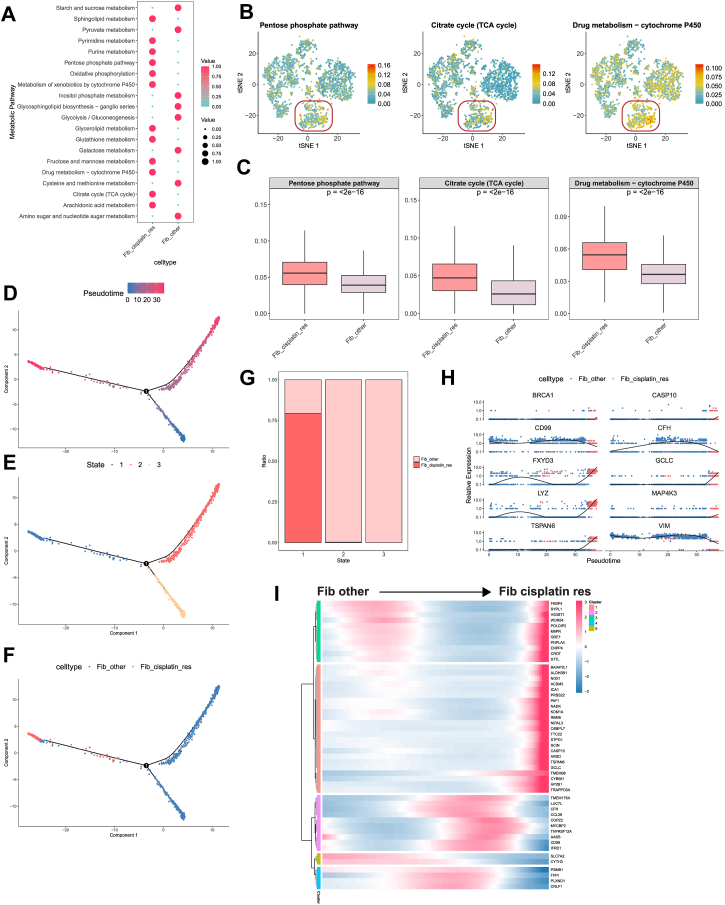
**Metabolic reprogramming and differentiation trajectories of Fib_cisplatin_res in BC**. *A*, differential metabolic pathways of fibroblast subpopulations identified *via* scMetabolism analysis. *B*, t-SNE visualization showing the enrichment distribution of two cell subpopulations in specific metabolic pathways. *C*, *boxplot* comparison of differential expression in specific metabolic pathways between the two subpopulations. *D*, pseudotime trajectory analysis of fibroblast subpopulations using Monocle 2, illustrating transcriptional state changes. *E*, pseudotime distribution of fibroblast subpopulations in different states. *F*, distribution of Fib_cisplatin_res and Fib_other subpopulations in pseudotime analysis. *G*, proportions of the two fibroblast subpopulations in various states. *H*, expression trends of key transcription factors in fibroblast subpopulations during pseudotime analysis. *I*, *heatmap* showing dynamic transcription factor expression changes in fibroblast subpopulations. BC, bladder cancer; Fib_cisplatin_res, fibroblast cisplatin–resistant subpopulation; Fib_other, fibroblast nonresistant subpopulation; t-SNE, t-distributed stochastic neighbor embedding.

Metabolic pathway enrichment analysis of scRNA-Seq data, performed using scMetabolism, revealed active metabolic activity in Fib_cisplatin_res cells across various pathways, particularly glycolysis/gluconeogenesis, the tricarboxylic acid (TCA) cycle, and cytochrome P450 pathways associated with drug metabolism (Fig. 6*A*). These enriched pathways suggest that cisplatin-resistant cells may acquire a survival advantage through metabolic reprogramming. Notably, Fib_cisplatin_res cells exhibited higher metabolic activity in glycolysis, the TCA cycle, and drug metabolism pathways compared with other cells (Fig. 6, *B* and *C*). Further analysis of scRNA-Seq data showed that these pathways scored higher in Fib_cisplatin_res cells than in Fib_other cells, indicating that resistant cells may enhance glycolysis and the TCA cycle to meet the energy demands of rapid growth and proliferation (62). In addition, the cytochrome P450 pathway, which is critical for drug metabolism, demonstrated elevated activity in resistant cells, likely contributing to their detoxification and tolerance mechanisms against cisplatin (63). These findings highlight the pivotal role of metabolic reprogramming in Fib_cisplatin_res cells. By intensifying glycolysis, the TCA cycle, and drug metabolism pathways, resistant cells can effectively counter cisplatin treatment and sustain their survival.

Using Monocle for pseudotime analysis, the developmental trajectory of BC fibroblasts was successfully constructed, tracing their progression from a cisplatin-sensitive state (Fib_other) to a cisplatin-resistant state (Fib_cisplatin_res) (Fig. 6, *D*–*F*). The results revealed that Fib_cisplatin_res cells predominantly occupied the later stages of the pseudotime trajectory, indicating their differentiation and evolution into a resistant cell population. This finding was further validated by state distribution analysis, where Fib_cisplatin_res cells were primarily located at the trajectory's endpoint (Fig. 6*G*). In contrast, Fib_other cells were more frequently observed in the early and intermediate stages, reflecting their higher sensitivity to cisplatin treatment. During the pseudotime progression, significant changes were observed in the expression of key transcription factors, including BRCA1, CASP10, CFH, FXYD3, GCLC, LYZ, MAP4K3, TSPAN6, and VIM (Fig. 6*H*). BRCA1, known to enhance DNA repair mechanisms, plays a critical role in mitigating cisplatin-induced damage and is strongly associated with chemoresistance to platinum-based therapies in breast cancer (64). Similarly, CASP10 contributes to resistance by regulating apoptotic pathways, enabling resistant cells to evade cisplatin-induced cell death (65). CFH regulates the complement system and plays a role in immune evasion mechanisms (66). FXYD3 maintains cellular electrolyte balance during stress responses, enhancing resistance to cisplatin-induced stress (56). GCLC counteracts cisplatin-induced oxidative stress by enhancing glutathione metabolism (67). LYZ may modulate inflammatory responses and immune activity under various conditions (44). MAP4K3 regulates cell survival *via* the mTOR signaling pathway, aiding cancer cells in adapting to chemotherapy-induced stress (68). TSPAN6 and VIM are associated with cell migration and invasion (69, 70). The upregulation of these transcription factors in Fib_cisplatin_res cells may be linked to biological processes, such as DNA repair, antiapoptosis, immune evasion, antioxidative responses, cell migration, and invasion. Heatmap analysis (Fig. 6*I*) revealed the expression patterns of key genes during the transition of BC fibroblasts from cisplatin sensitivity to resistance. Compared with cisplatin-sensitive cells, genes upregulated in resistant cell populations are likely involved in proliferation, survival, DNA repair, and stress response mechanisms. The upregulation of HS3ST1 suggests its involvement in extracellular matrix remodeling and TME reconstruction, enhancing cell survival and cisplatin resistance (71). Elevated POLDIP2 expression indicates its critical role in the DDR pathway, enabling cells to cope with cisplatin-induced DNA damage (72). Increased GCLC expression is associated with enhanced glutathione metabolism, likely contributing to resistance by strengthening antioxidative responses against cisplatin-induced oxidative stress (73). SCIN upregulation modulates cytoskeletal proteins, promoting cell migration and invasion (74). These genes likely work synergistically to regulate cell migration, invasion, stress responses, and DNA repair, collectively driving cisplatin resistance. These findings provide valuable insights into the specific roles of these genes in BC resistance mechanisms, offering important clues for future research.

### *In vitro* functional validation: SPINK1 and the MIF signaling axis drive cisplatin resistance in BC

**Figure 7:**
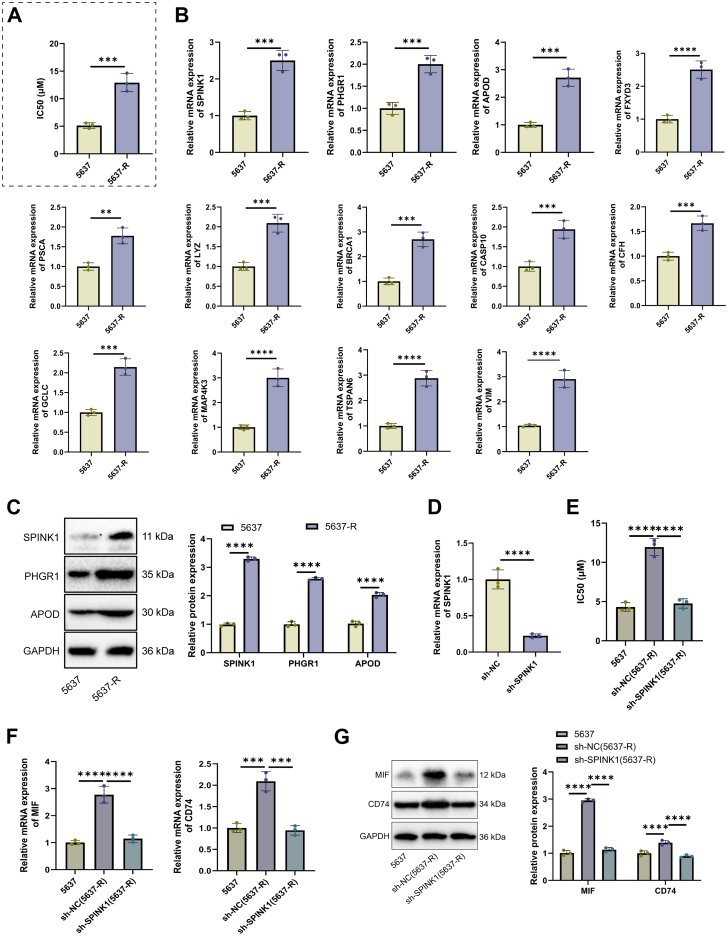
**Establishment of a cisplatin-resistant BC cell line and functional validation of the *SPINK1* gene**. *A*, MTT assay measuring the IC_50_ values of parental 5637 cells and cisplatin-resistant 5637-R cells. *B*, RT–quantitative PCR (qPCR) analysis showing mRNA expression levels of the resistance-associated genes (*SPINK1*, *PHGR1*, *APOD*, *FXYD3*, *PSCA*, *LYZ*, *BRCA1*, *CASP10*, *CFH*, *GCLC*, *MAP4K3*, *TSPAN6*, and *VIM*) in parental 5637 and 5637-R cells. *C*, Western blot analysis and quantitative densitometric evaluation of cisplatin resistance–associated proteins (SPINK1, PHGR1, and APOD) in parental 5637 and 5637-R cells. *D*, RT–qPCR validation of SPINK1 knockdown efficiency in the resistant strain. *E*, MTT assay assessing changes in cisplatin IC_50_ values in 5637-R cells following SPINK1 knockdown. *F*, RT–qPCR detection of changes in *MIF* and *CD74* mRNA expression after SPINK1 knockdown. *G*, Western blot analysis and quantification of MIF and CD74 protein expression following SPINK1 knockdown. Data are presented as mean ± SD, n = 3. ∗*p* < 0.05, ∗∗*p* < 0.01, ∗∗∗*p* < 0.001, and ∗∗∗∗*p* < 0.0001. BC, bladder cancer; MIF, macrophage migration inhibitory factor; MTT, 3-(4,5-dimethylthiazol-2-yl)-2,5-diphenyltetrazolium bromide.

To validate the cisplatin resistance–associated molecular features identified *via* bioinformatic analyses, we first established an *in vitro* cisplatin-resistant BC cell model. Human BC 5637 cells were subjected to a stepwise increasing concentration gradient of cisplatin. After 8 months of continuous induction, a resistant subline capable of stable proliferation in 1 μM cisplatin was successfully established and designated as 5637-R. 3-(4,5-Dimethylthiazol-2-yl)-2,5-diphenyltetrazolium bromide (MTT) assays demonstrated that, compared with parental 5637 cells, the IC_50_ value of cisplatin was significantly elevated in 5637-R cells, confirming the successful establishment of the resistant phenotype (Fig. 7*A*). Subsequent RT–quantitative PCR (qPCR) analysis revealed that, relative to parental cells, mRNA expression levels of *SPINK1*, *PHGR1*, *APOD*, *FXYD3*, *PSCA*, *LYZ*, *BRCA1*, *CASP10*, *CFH*, *GCLC*, *MAP4K3*, *TSPAN6*, and *VIM* were markedly upregulated in 5637-R cells. Notably, *SPINK1*, *PHGR1*, and *APOD* exhibited the most pronounced upregulation (Fig. 7*B*). To further verify the biological relevance of these key factors, Western blot analysis was performed. The results indicated that protein levels of SPINK1, PHGR1, and APOD were significantly elevated in 5637-R cells compared with parental cells. These findings align with survival analyses across multiple cancer types, where resistance-associated DEGs (such as *SPINK1*, *PHGR1*, and *APOD*) correlated with poor prognosis (Fig. 7*C*). Collectively, these results validate the reliability of our bioinformatic findings at both cellular and molecular levels.

Given that *SPINK1* exhibited the most significant overexpression in the resistant strain, we conducted gene knockdown experiments to confirm its core driver role in maintaining the resistant phenotype. Transfection of 5637-R cells with sh-SPINK1 resulted in effective suppression of *SPINK1* expression, as confirmed by RT–qPCR (Fig. 7*D*). MTT assays demonstrated that, compared with the sh-NC group, SPINK1 knockdown significantly restored cisplatin sensitivity in 5637-R cells, reducing the IC_50_ value to levels comparable to those of parental 5637 cells (Fig. 7*E*). Further mechanistic investigations revealed that SPINK1 silencing led to a marked downregulation of *MIF* and its receptor *CD74* at the mRNA level (Fig. 7*F*). Western blot analysis corroborated these findings, showing a synchronous reduction in MIF and CD74 protein expression in sh-SPINK1-treated 5637-R cells compared with the sh-NC group (Fig. 7*G*). These data suggest that SPINK1 functions not merely as a resistance marker but as a functional driver gene positioned upstream of the MIF signaling axis.

**Figure 8:**
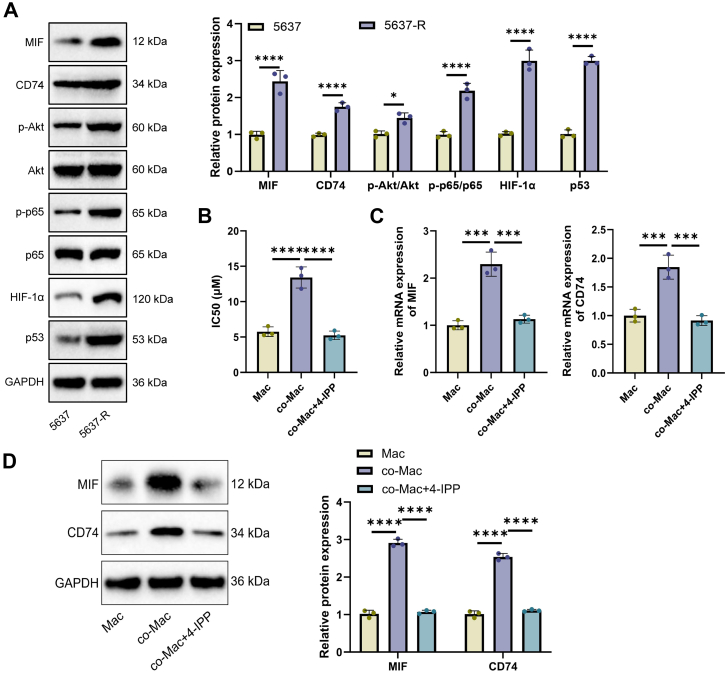
**Protective interaction between cisplatin-resistant tumor cells and macrophages mediated by the MIF signaling axis**. *A*, Western blot analysis and quantitative densitometric evaluation of MIF and related signaling pathways (Akt, p65, HIF-1, and p53) in parental 5637 cells and cisplatin-resistant 5637-R cells. *B*, MTT assay measuring the IC_50_ values of macrophages in each group. *C*, RT–quantitative PCR validation of MIF and CD74 gene expression changes in macrophages across groups. *D*, Western blot detection of MIF and CD74 protein expression in macrophages along with quantitative analysis of protein levels. Data are presented as mean ± SD, n = 3, ∗*p* < 0.05, ∗∗∗*p* < 0.001, and ∗∗∗∗*p* < 0.0001. MIF, macrophage migration inhibitory factor; MTT, 3-(4,5-dimethylthiazol-2-yl)-2,5-diphenyltetrazolium bromide.

Building on these findings and the single-cell communication analysis, we further investigated whether resistant cells interact with the microenvironment *via* the SPINK1-regulated MIF signaling axis. Initially, we assessed the activation status of the MIF signaling pathway in the resistant strain. Western blot results indicated a distinct upregulation of MIF and its receptor CD74 in 5637-R cells, accompanied by the activation of downstream pathways, including Akt, p65, HIF-1, and p53 (Fig. 8*A*). To validate intercellular interactions, a Transwell coculture system was established. MTT assays revealed that, compared with macrophages cultured alone, macrophages cocultured with resistant cells displayed enhanced cisplatin tolerance. However, this protective effect was reversed by the addition of the MIF inhibitor 4-iodo-6-phenylpyrimidine (4-IPP) (Fig. 8*B*). Both RT–qPCR and Western blot analyses confirmed that resistant cells induce the upregulation of MIF and CD74 expression in macrophages, a process significantly suppressed by 4-IPP treatment (Fig. 8, *C* and *D*). These results confirm that resistant cells establish a protective interaction with macrophages through the SPINK1–MIF–CD74 signaling axis and that targeting this axis can disrupt the resistance-promoting microenvironment.

## Discussion

Cisplatin resistance in BC poses a significant challenge in clinical treatment, with its intricate molecular mechanisms remaining a focal point of research. Through single-cell omics integration analysis, this study is the first to reveal, at single-cell resolution, the critical links between cellular heterogeneity in the TME and cisplatin resistance in BC. The findings highlight significant heterogeneity among epithelial cells and fibroblasts during the development of resistance, particularly in metabolic reprogramming processes involving glycolysis, DDR, and drug metabolism pathways. Moreover, intercellular communication analysis identified complex signaling networks between resistant cell subpopulations, immune cells, and fibroblasts, with pathways such as MIF, THBS, MHC-II, and FN1 exhibiting high activity. These insights provide a multidimensional perspective on how the TME drives cisplatin resistance in BC through cellular heterogeneity and signaling networks, offering a deeper understanding of this complex process.

Cellular heterogeneity is a central topic in TME research (75). Previous studies have predominantly focused on bulk gene expression analyses, which fail to delineate the specific roles of individual cell subpopulations in drug resistance (76, 77, 78). In this study, we utilized scRNA-Seq to refine the characterization of heterogeneity within epithelial cells and fibroblasts in the BC TME, particularly identifying the drug resistance–associated subpopulations Epi_cisplatin_res and Fib_cisplatin_res. Unlike the generalized descriptions of drug-resistant cells at the population level found in previous literature, this study reveals significant differences in gene expression and metabolic activity among these subpopulations through single-cell techniques. These findings provide a more precise framework for understanding drug resistance and expand our knowledge of functional differentiation among cells within the TME.

Metabolic reprogramming is a key feature of tumor resistance mechanisms. Previous studies have demonstrated that enhanced glycolysis and drug metabolism–related pathways play critical roles in cisplatin resistance (79, 80, 81). However, these studies often focus on the overall metabolic characteristics of bulk samples, overlooking the metabolic heterogeneity of distinct cellular subpopulations within the TME (82, 83, 84). Using scMetabolism analysis, this study is the first to reveal significant differences in glycolysis, DDR, and drug metabolism pathways between resistant epithelial cells and fibroblasts. Notably, Epi_cisplatin_res cells exhibit high levels of glycolysis and DNA repair capacity, which are closely associated with the development of resistance. These findings not only provide new insights into the role of metabolic reprogramming in resistance mechanisms but also offer potential targets for therapies aimed at metabolic pathways.

Intercellular communication networks play a critical role in the TME. Previous studies have primarily focused on signaling pathways between immune cells, with limited attention given to communication between drug-resistant cell subpopulations and other cell types (85, 86, 87, 88, 89). Through integrated analyses using CellChat and CellPhoneDB, this study reveals a complex signaling network between drug-resistant cell subpopulations, immune cells, and fibroblasts. These communication patterns exhibit significant activity in signaling pathways, such as MIF, THBS, MHC-II, and FN1, which are strongly associated with drug resistance. Moreover, the study highlights that these pathways not only directly influence drug-resistant cells but also may indirectly promote drug resistance by modulating immune cell functions. These findings provide important insights into the role of intercellular communication in the development of drug resistance.

Dynamic analysis is a crucial tool for understanding cellular behavior changes within the TME (90). Previous studies predominantly employed static analysis methods, which are insufficient for capturing the dynamic evolution during the development of drug resistance. In this study, pseudotime analysis using Monocle was utilized to uncover the dynamic transition trajectory of fibroblasts from cisplatin-sensitive to -resistant states. The results demonstrated that fibroblasts undergo progressive changes in gene expression and metabolic activity during the acquisition of drug resistance, particularly through the activation of the FN1 signaling pathway and drug metabolism–related pathways. These findings provide new insights into the potential evolutionary trajectories underlying the development of drug resistance and highlight promising therapeutic intervention targets, while also offering a theoretical foundation for strategies aimed at reversing chemoresistance.

This study also identified several DEGs associated with drug resistance (*e*.*g*., SPINK1, PHGR1, and APOD) and validated their prognostic value across various cancers through survival analysis using the TCGA database. The results indicated that these genes are strongly associated with poor prognosis and may serve as potential biomarkers for BC. Furthermore, the differential expression of these genes across cancer types highlights their potential utility in tumor-specific therapies. These findings support the development of personalized treatment strategies based on molecular biomarkers and expand their potential clinical applications.

This study systematically elucidates the molecular mechanisms underlying cisplatin resistance in BC, with a particular focus on the roles of cellular heterogeneity, metabolic reprogramming, and intercellular communication within the TME. These findings offer novel insights into the complex biological processes driving resistance and provide a foundation for developing targeted therapeutic strategies aimed at metabolic pathways and signaling networks. Clinically, the results of this study could aid in predicting patient responses to chemotherapy, guiding personalized treatment plans, and ultimately improving therapeutic outcomes and patient survival rates.

Despite these significant advancements, the study has several limitations. First, the data primarily originate from publicly available databases, which may not fully represent the diversity of patient populations. Second, further experimental validation is needed, particularly functional assays in *in vivo* models. In addition, the study does not incorporate integrative analyses of other omics data, such as proteomics or epigenomics, which may limit a comprehensive understanding of resistance mechanisms. Future research should aim to expand sample sizes, integrate omics data, and explore novel experimental models to validate and extend these findings. Furthermore, the development of more efficient targeted therapies and early diagnostic tools will offer improved treatment options and survival prospects for BC patients. This study provides a solid foundation for resistance mechanism research and therapeutic strategy development, contributing significantly to the advancement of precision medicine in BC.

## Conclusion

**Figure 9:**
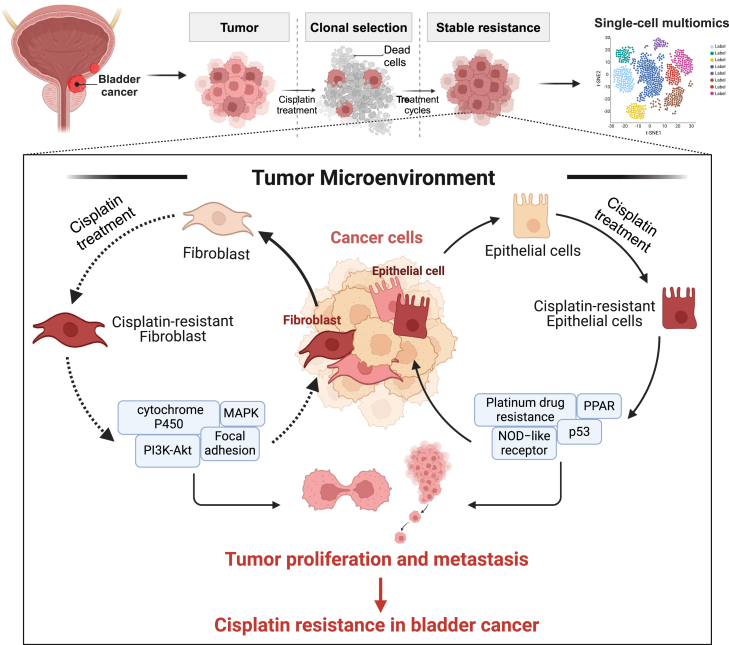
**A schematic representation illustrating the molecular mechanisms of cisplatin resistance in BC, as revealed through integrated single-cell omics analysis of cellular heterogeneity within the TME**. BC, bladder cancer; TME, tumor microenvironment.

In conclusion, this study systematically elucidates the cellular heterogeneity and molecular mechanisms underlying cisplatin resistance in the TME of BC through integrated single-cell omics analysis. For the first time, we identified and characterized the Epi_cisplatin_res and Fib_cisplatin_res in BC, highlighting their critical roles in the development of cisplatin resistance (Fig. 9). Detailed analyses of the metabolism, gene expression, and developmental trajectories of these cisplatin-resistant subpopulations revealed significant metabolic reprogramming and transcriptional remodeling in pathways such as glycolysis, DDR, and drug metabolism. These findings suggest that these subpopulations may acquire resistance by enhancing energy metabolism and stress adaptation to chemotherapeutic agents. Furthermore, key DEGs associated with resistance (*e*.*g*., SPINK1, PHGR1, and APOD) are linked to poor prognosis across various cancers, indicating their potential clinical value as prognostic biomarkers and therapeutic targets in BC and beyond. Cell–cell communication analysis further uncovered a complex signaling network between resistant subpopulations and immune cells and fibroblasts within the TME, providing novel insights into the mechanisms of cisplatin resistance in BC. Future studies should focus on exploring the molecular characteristics and immune evasion mechanisms of these resistant subpopulations. Clinical validation of these findings is essential to facilitate the development of novel therapeutic strategies and targeted drugs for overcoming cisplatin resistance in BC.

## Experimental procedures

### Data collection

Single-cell transcriptomic data were obtained from the Gene Expression Omnibus database, including six primary tumor samples and one adjacent nontumorous tissue sample from six patients with BC (GSE135337). The six primary tumor samples were labeled as GSM4006644, GSM4006645, GSM4006646, GSM4006647, GSM4006648, and GSM4751267, whereas the adjacent tissue sample was labeled as GSM5329919. In addition, we collected one cisplatin-sensitive sample (Sen, GSM5751919) and one cisplatin-resistant sample (Res, GSM5751918) derived from human BC in the GSE192575 dataset.

### Processing and annotation of scRNA-Seq data

To ensure data integrity and consistency, we conducted rigorous quality control to exclude low-quality cells. The screening criteria included cells with fewer than 200 or more than 6000 expressed genes, unique molecular identifiers exceeding 25,000, or a mitochondrial gene proportion greater than 10%. Valid cells were retained for subsequent analysis. We utilized the R package Seurat (version 4.1.0) for data normalization and applied the Harmony tool (version 1.0.0) to correct batch effects. The FindVariableFeatures function was employed to identify the top 2000 highly variable genes, followed by dimensionality reduction using principal component analysis to extract key features. To visualize the overall structure of the cellular population, we performed dimensionality reduction using uniform manifold approximation and projection and t-SNE methods. For cell clustering, the FindNeighbors and FindClusters functions were used with a resolution parameter set to 0.6, enabling the identification of distinct cell subpopulations. Cell type annotation was performed using the SingleR package (version 1.8.1), which compared the data with the Human Cell Landscape to accurately identify major cell types, including epithelial cells, immune cells, and fibroblasts. The annotation results were further validated using canonical marker genes from published literature, ensuring reliable identification of each cell subpopulation.

### Detection of cell cluster–specific genes

To identify genes specifically expressed in different cell clusters, we conducted DEG analysis using the FindAllMarkers function in the Seurat package (version 4.1.0). DEGs were assessed within each cell cluster using the Wilcoxon's rank-sum test. The criteria for defining DEGs included expression in more than 25% of cells within a cluster, a log2 FC greater than 0.25 compared with the background, and a false discovery rate less than 0.05. For specific cell types, the FindMarkers function was used to compare cisplatin-sensitive and cisplatin-resistant groups, identifying key genes associated with cisplatin resistance.

### Analysis of intercellular communication in the TME

The intercellular communication patterns within the TME were analyzed using the CellChat tool (version 1.1.3), focusing on interactions among epithelial cells, fibroblasts, and macrophages. Standardized gene expression data were input into CellChat, followed by preprocessing steps, such as identifyOverExpressedGenes, identifyOverExpressedInteractions, and projectData, ensuring data accuracy. The functions aggregateNet, computeCommunProbPathway, and computeCommunProb were employed to calculate the information flow strength and communication probability of L–R pairs between different cell populations. These calculations were used to construct the CellChat network within the TME, detailing the communication probabilities and information flow intensities between cell groups. To further investigate the L–R interactions between epithelial cells and other cell types in their microenvironment, the CellPhoneDB tool (version 2.1.4) was utilized. The standardized gene expression matrix was input into CellPhoneDB, where data preprocessing identified L–R interaction pairs among cell types. Significant molecular pairs (*p* < 0.05) and their interaction strengths were identified, highlighting potential key players in the development of cisplatin resistance.

### Identification of cisplatin-resistant subpopulations

To identify epithelial cisplatin–resistant subpopulation (Epi_cisplatin_res) in BC, an unsupervised clustering analysis of epithelial cells was performed. The preprocessed epithelial cell expression matrix was clustered using the Seurat package (version 4.1.0), with the resolution parameter set to 0.6 to ensure the identification of distinct and heterogeneous cell subgroups. Subsequently, the DDRs of each epithelial cell were quantified using the AUCell (Area Under the Curve Cell, v1.14.0) tool. AUCell evaluates the activity of gene sets within single cells, providing a quantitative assessment of DDR-related gene expression. Based on the DDRs, cluster 4 was defined as the Epi_cisplatin_res, whereas the remaining epithelial cell clusters were categorized as epithelial-nonresistant subpopulation (Epi_other).

To further explore the heterogeneity among fibroblast subpopulations, unsupervised clustering was also conducted for fibroblasts. The Seurat FindClusters function was employed for clustering analysis, and marker gene expression patterns of different cell groups were compared. Cluster 2 was identified as the Fib_cisplatin_res, whereas the remaining fibroblast subpopulations were designated as fibroblast nonresistant subpopulation (Fib_other).

### Differential gene and functional enrichment analysis

To further validate the association between these subpopulations and cisplatin resistance, DEG analysis was conducted using the FindMarkers function to compare cisplatin-resistant subpopulations with other cellular subpopulations. Key genes associated with cisplatin resistance were identified. The results were visualized using a volcano plot, with the selection criteria set as log2 FC >1 or <−1 and a corrected *p* value threshold of *q* < 0.05. Enrichment analysis of the DEGs was performed using the R package clusterProfiler (version 4.2.2). In addition, the expression of these DEGs was mapped onto cellular expression data to observe their distribution within the resistant subpopulations.

### Survival analysis

Transcriptome data and corresponding clinical information for ACC and KIRC patients were downloaded from the TCGA database (https://portal.gdc.cancer.gov/). The dataset included RNA-Seq data (RNA-Seq by Expectation Maximization values) from tumor and normal tissues of hundreds of cancer patients, provided in Fragments Per Kilobase of exon per Million reads mapped format. After standardization, the RNA-Seq data were utilized for subsequent differential expression and survival analyses. Clinical information for the samples included survival time, age, pathological stage, and other relevant variables.

To evaluate the relationship between DEG levels and prognosis in ACC and KIRC patients, Kaplan–Meier survival analysis was conducted using the R packages survival (version 3.2-11) and survminer (version 0.4.9). Patients were divided into high-expression and low-expression groups based on the median expression level of the DEGs. Survival curves for the two groups were compared using the log-rank test to determine the association between gene expression levels and overall survival.

### scMetabolism

To investigate the phenomenon of metabolic reprogramming in drug-resistant cells, the R package scMetabolism (version 0.2.1) was used to perform metabolic pathway enrichment analysis on scRNA-Seq data. The standardized single-cell gene expression matrix was first input into scMetabolism for preprocessing, which included calculating the expression levels of metabolic genes in each cell to ensure data accuracy and consistency. Metabolic activity in individual cells was quantified using scMetabolism, and the activity levels of metabolic pathways were assessed with the AUCell method. Pathways showing significant activity in drug-resistant cells were identified and subjected to the KEGG functional enrichment analysis to determine their biological significance.

### Pseudotime analysis

To investigate the developmental trajectory of BC epithelial cells and fibroblasts transitioning from cisplatin sensitivity to resistance, pseudotime analysis was performed using the R package "Monocle" (version 2.22.0) on scRNA-Seq data. The standardized and annotated single-cell expression matrix was first input into Monocle for preprocessing, which included selecting highly variable genes, removing lowly expressed genes, and performing dimensionality reduction to ensure data accuracy and integrity. Dimensionality reduction was conducted using the ReduceDimension function, with DDRTree chosen as the method, constructing pseudotime trajectories for epithelial cells and fibroblasts. To further explore the developmental and evolutionary relationships of cisplatin-resistant subpopulations (Epi_cisplatin_res and Fib_cisplatin_res), key transcriptional nodes within the pseudotime trajectories were identified and annotated. The plot_cell_trajectory function was used to visualize the pseudotime trajectories, and Monocle's BEAM analysis was employed to select genes with *q* values <1e-4. Dynamic gene expression patterns were visualized using the plot_genes_branched_heatmap function.

### Cell culture and establishment of cisplatin-resistant cell line

Human BC 5637 cells (CL-0002; Wuhan PriCells Biotechnology Co, Ltd) were cultured in RPMI1640 medium (11875093, Gibco) supplemented with 10% fetal bovine serum (10099141C; Gibco) in a humidified incubator at 37 °C with 5% CO_2_. Cells in the logarithmic growth phase were used to establish a cisplatin-resistant cell line using a stepwise dose-escalation method. The parental 5637 cells were exposed to gradually increasing concentrations of cisplatin (15663-27-1; Sigma–Aldrich) ranging from 2 nM to 1 μM over an 8-month period. The initial concentration of 2 nM was applied for 7 days, and once stable cell growth was observed, the concentration was progressively increased up to 1 μM. During this period, the drug-containing medium was replaced every 2 to 3 days to maintain cell viability. Ultimately, a cisplatin-resistant cell line capable of stable growth in 1 μM cisplatin, designated 5637-R, was successfully established (91).

### Cell proliferation assay

Cells were seeded into 96-well plates at a density of 5000 cells per well and incubated for 24 h, followed by treatment with 1 μM cisplatin for 72 h. Subsequently, 20 μl of MTT solution (M5655; Sigma–Aldrich; 5 mg/ml dissolved in PBS) was added to each well and incubated at 37 °C for 2 h. After removing the supernatant, 150 μl of dimethyl sulfoxide (D2650; Sigma–Aldrich) was added to dissolve the formazan crystals by gentle shaking. Absorbance was measured at 490 nm using a microplate reader (ELX-800; Bio-Tek). The IC_50_ value (the drug concentration that reduces cell viability to 50% of the control group) was calculated using GraphPad 5 software (GraphPad Software, Inc.) (92).

### RT–qPCR analysis of gene expression

Total RNA was extracted using TRIzol (Invitrogen) according to the manufacturer’s instructions, with all procedures performed under RNase-free conditions. After centrifugation, the aqueous phase was transferred to a new tube, precipitated with isopropanol, washed with 75% ethanol, and dissolved in nuclease-free water. To eliminate residual genomic DNA, RNase-free DNase I (Thermo) was added prior to loading and incubated for 15 min, followed by heat inactivation or column purification. RNA quality was assessed using a NanoDrop spectrophotometer, and samples with absorbance at 260 nm/absorbance at 280 nm values between 1.9 and 2.1 and absorbance at 260 nm/absorbance at 230 nm >1.8 were considered acceptable. Integrity was further evaluated using an Agilent 2100 Bioanalyzer, and only samples with an RNA integrity number ≥7.0 were included in subsequent analyses. RT was performed using HiScript III or Maxima First Strand Complementary DNA (cDNA) Synthesis Kits (Vazyme/Thermo), primed with a mixture of oligo(dT) and random hexamers. A total of 500 to 1000 ng RNA was used per reaction, and cDNA synthesis was conducted with the program: 25 °C for 5 min → 50 °C for 15 to 30 min → 85 °C for 5 min. The resulting cDNA was diluted 1:5 to 1:10 and stored at −20 °C for short-term use or −80 °C for long-term storage. No-RT controls were included to monitor genomic DNA contamination. Primers were designed based on the National Center for Biotechnology Information RefSeq sequences, spanning exon–exon junctions and avoiding SNP-rich regions, with amplicon lengths between 80 and 200 bp. Primer specificity was validated by gel electrophoresis to ensure a single band, followed by sequencing confirmation. All primer sequences are listed in Table 1.

**Table 1:** RT–qPCR primer sequences

Gene	Primer sequence
SPINK1 (human)	Forward: 5′-CACCTGGCTCCTTTCACCTT-3′
	Reverse: 5′-GTTCTCAGCAAGGCCCAGAT-3′
PHGR1 (human)	Forward: 5′-GAGAATTGCCCAGCTGACCT-3′
	Reverse: 5′-TGGCCTCTAGGAGGCTGTTT-3′
APOD (human)	Forward: 5′-GATCCTGGCCACCGACTATG-3′
	Reverse: 5′-ACAGCAGGTCAGCAACAAGT-3′
MIF (human)	Forward: 5′-GTGGTGTCCGAGAAGTCAGG-3′
	Reverse: 5′-TTGCTGTAGGAGCGGTTCTG-3′
CD74 (human)	Forward: 5′-GGCAACATGACAGAGGACCA-3′
	Reverse: 5′-TCCAAGGGTGACGAAAGAGC-3′
FXYD3 (human)	Forward: 5′-GAAGTGAGGTTTGCTTAGGGC-3′
	Reverse: 5′-TCTCGGGGTAAGTGAGAGGG-3′
PSCA (human)	Forward: 5′-GTGACACCGACTTGTGCAAC-3′
	Reverse: 5′-TGCGTTAGGATGTGCCTCAG-3′
LYZ (human)	Forward: 5′-TTTCTGTTACGGTCCAGGGC-3′
	Reverse: 5′-CCATGCCACCCATGCTCTAA-3′
BRCA1 (human)	Forward: 5′-GAGGAACGGGCTTGGAAGAA-3′
	Reverse: 5′-TGCATGGTATCCCTCTGCTG-3′
CASP10 (human)	Forward: 5′-TCTTGGAAGCCTTACCGCAG-3′
	Reverse: 5′-ATCTCTTCACCTTGGCAGGC-3′
CFH (human)	Forward: 5′-ATGGATGGTCAGCTCAACCC-3′
	Reverse: 5′-GAACCAGTGGTGCTTCCAGT-3′
GCLC (human)	Forward: 5′-ACTTCATTTCCCAGTACCTTAACA-3′
	Reverse: 5′-CCGGCTTAGAAGCCCTTGAA-3′
MAP4K3 (human)	Forward: 5′-ATGAACCCCGGCTTCGATTT-3′
	Reverse: 5′-CCAGTGTTAACATTCCGTGCC-3′
TSPAN6 (human)	Forward: 5′-TTCTCGCCTACTGCCTCTCT-3′
	Reverse: 5′-ATCACCATCTGGCAACCCTG-3′
VIM (human)	Forward: 5′-CTCTGGCACGTCTTGACCTT-3′
	Reverse: 5′-TTGCGCTCCTGAAAAACTGC-3′
GAPDH (human)	Forward: 5′-GTCTCCTCTGACTTCAACAGCG-3′
	Reverse: 5′-ACCACCCTGTTGCTGTAGCCAA-3′

Real-time qPCR was performed using SYBR Green–based detection reagents (ChamQ SYBR qPCR Master Mix or PowerUp SYBR Green Master Mix) on either a QuantStudio 6 or LightCycler 480 instrument. The standard cycling protocol was as follows: initial denaturation at 95 °C for 2 min, followed by 40 cycles of 95 °C for 15 s and 60 °C for 30 s (with minor adjustments to the annealing temperature as required by primer design). At the end of amplification, a melt curve analysis (65–95 °C, 0.5 °C increments) was conducted to confirm the presence of a single specific peak. Each sample was analyzed in triplicate (technical replicates), and a no-template control was included in every assay. GAPDH served as the internal reference gene. Relative gene expression levels were calculated using the 2ˆ−ΔΔCt method (or an efficiency-corrected formula when appropriate). Specifically, ΔCt = Ct(target) − Ct(reference) and ΔΔCt = ΔCt(treated) − ΔCt(control). Expression levels were normalized to the control group, which was set to 1 (91).

### Western blot analysis

Cells were lysed on ice for 30 min using radioimmunoprecipitation assay buffer containing protease and phosphatase inhibitors (catalog no.: 89900; Thermo Fisher Scientific), with vortexing every 5 min to enhance lysis efficiency. Lysates were centrifuged at 12,000*g* for 15 min at 4 °C to remove insoluble debris, and the supernatant was collected. Protein concentrations were determined using a BCA Protein Assay Kit (catalog no.: 23227; Thermo Fisher Scientific) to ensure equal loading across samples. For each sample, 20 to 40 μg of protein was mixed with 4× Laemmli sample buffer and denatured at 95 °C for 5 min before loading onto SDS-PAGE gels (8–12% resolving gel, 5% stacking gel), with the gel concentration selected based on the molecular weight of the target proteins.

Following electrophoresis, proteins were wet-transferred onto polyvinylidene fluoride membranes (catalog no.: IPVH00010; Millipore), which were preactivated in methanol for 1 min prior to transfer. Protein transfer was performed at a constant current of 300 mA for 60 to 90 min under ice-bath conditions to prevent protein denaturation. Membranes were then blocked with 5% nonfat milk in Tris-buffered saline with Tween-20 for 1 h and incubated overnight at 4 °C with the following primary antibodies: anti-SPINK1 (ab206294, 1:1000 dilution; Abcam), anti-PHGR1 (ab181131, 1:1000 dilution; Abcam), anti-APOD (ab108191, 1:1000 dilution; Abcam), anti-MIF (ab175189, 1:1000 dilution; Abcam), anti-CD74 (ab270265, 1:1000 dilution; Abcam), anti–phospho-Akt (ab38449, 1:1000 dilution; Abcam), anti-Akt (ab32505, 1:1000 dilution; Abcam), anti–phospho-p65 (ab76302, 1:1000 dilution; Abcam), anti-p65 (ab32536, 1:1000 dilution; Abcam), anti–HIF-1α (ab179483, 1:1000 dilution; Abcam), anti-p53 (ab32049, 1:1000 dilution; Abcam), and anti-GAPDH (ab22555, 1:1000 dilution; Abcam) as the internal control. On the following day, membranes were washed three times with Tris-buffered saline with Tween-20 (10 min each) and incubated with the corresponding horseradish peroxidase–conjugated secondary antibody (ab6721, 1:1000 dilution; Abcam) for 1 h at room temperature. Protein bands were visualized using an ECL detection reagent (catalog no.: 32106; Thermo Fisher Scientific) and captured with a chemiluminescence imaging system (Bio-Rad ChemiDoc XRS+). Band intensities were quantified using ImageJ software, and target protein signals were normalized to GAPDH to calculate relative expression levels. To ensure reproducibility, each group included at least three independent biological replicates (91).

### Transfection assay

Cells were digested with trypsin and seeded into six-well plates, ensuring a confluency of 60% to 70% on the day of transfection. The shRNA sequence targeting SPINK1 (GCCAAATGTTACAATGAACTT) was cloned into the pLKO.1-puro lentiviral expression vector (Merck KGaA) to generate the shSPINK1 plasmid, and a negative control plasmid containing a scrambled shRNA sequence (CAACAAGATGAAGAGCACCAA) was constructed in parallel. Transfections were performed using Lipofectamine 2000 (Life Technologies; Thermo Fisher Scientific, Inc) following the manufacturer’s instructions. 5637-R cells were seeded into 6-well plates at a density of 2.5 × 10^5^ cells per well in complete RPMI1640 medium. When cultures reached approximately 70% confluency, the medium was replaced with 2 ml of fresh complete medium. For each well, 2.5 μg of plasmid DNA and 5 μl of Lipofectamine 2000 were separately diluted in 100 μl of Opti-MEM medium (Life Technologies; Thermo Fisher Scientific, Inc) and incubated at room temperature for 5 min. The diluted DNA and Lipofectamine were then combined and allowed to stand for 20 to 30 min to form transfection complexes, which were subsequently added dropwise to the cells. After 6 to 8 h of transfection, the medium was replaced with fresh complete medium. At 48 to 72 h post-transfection, the knockdown efficiency of SPINK1 was assessed by qRT–PCR (92).

### Transwell coculture system

A Transwell coculture system with 8-μm pore inserts (3422; Corning) was used. Cisplatin-resistant BC cells were seeded into the upper chamber at a density of 2 × 10^4^ cells per well, whereas primary human macrophages (CP-H264; Wuhan PriCells Biotechnology Co, Ltd) were seeded into the lower chamber at 5 × 10^4^ cells per well. Cells were maintained in RPMI1640 medium supplemented with 10% serum. After 24 h of coculture, cisplatin (1 μM) was added simultaneously to both the upper and lower chambers for 48 h. In designated experimental groups, the MIF inhibitor 4-IPP (HY-110063; MedChemExpress; final concentration of 50 μM) was added to the coculture system for 48 h. Following treatment, macrophages from the lower chamber were collected for IC_50_ analysis, RT–qPCR, and Western blot assays (93).

### Statistical analysis

All experimental data are presented as mean ± SD. Comparisons between two groups were performed using two-tailed Student’s *t* tests, whereas multiple-group comparisons were conducted using one-way ANOVA, followed by Tukey’s post hoc test. For datasets that did not follow a normal distribution, the Mann–Whitney *U* test was applied. Correlation analyses were performed using either Pearson's or Spearman's methods, depending on data distribution. All statistical analyses were carried out using R (version 4.2.1) and GraphPad Prism (version 9.0), and differences were considered statistically significant at *p* < 0.05.

## Data availability

The datasets used or analyzed during the current study are available from the corresponding author on reasonable request.

## Supporting information

This article contains supporting information.

## Conflict of interest

The authors declare that they have no conflicts of interest with the contents of this article.
