# Supplementary material for: Single-cell insights into cisplatin resistance mechanisms in bladder cancer tumor microenvironment
Source: J Biol Chem. 2026 Feb 20;302(5):111304. doi: 10.1016/j.jbc.2026.111304 (PMC13092012; doi:10.1016/j.jbc.2026.111304)
Supplement: Supplementary Material [file mmc1.docx]

**
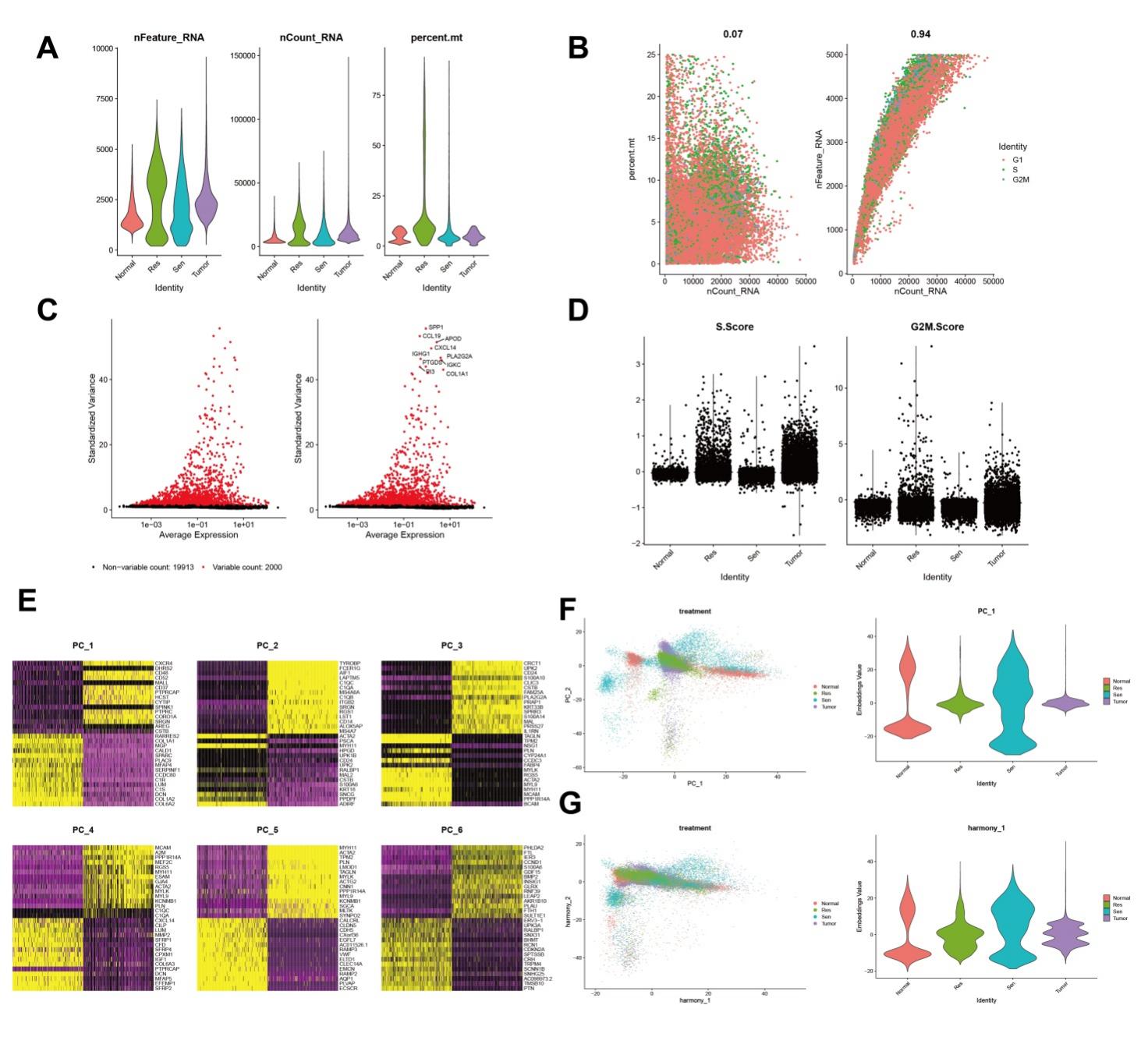
**

**Figure S1. Quality Control and Batch Effect Correction Analysis.**

Note: (A) Quality control results using the Seurat package, including assessments of cell data quality based on nFeature_RNA, nCount_RNA, and percent.mt metrics; (B) Correlation plots of nCount_RNA vs. percent.mt and nCount_RNA vs. nFeature_RNA, demonstrating sequencing depth and data quality; (C) Results of highly variable gene selection, showing the top 2000 highly variable genes; (D) Distribution of cell cycle scores for the S and G2M phases using the CellCycleScoring method; (E) Heatmap of genes associated with the first six principal components based on PCA; (F) Cell distribution in PC_1 and PC_2 before correction, evaluating batch effects; (G) Cell distribution in PC_1 and PC_2 after correction using the Harmony tool, demonstrating the effectiveness of batch effect correction. Groups include cisplatin-sensitive (Sen, n=1), cisplatin-resistant (Res, n=1), tumor (Tumor, n=6), and normal (Normal, n=1).

**
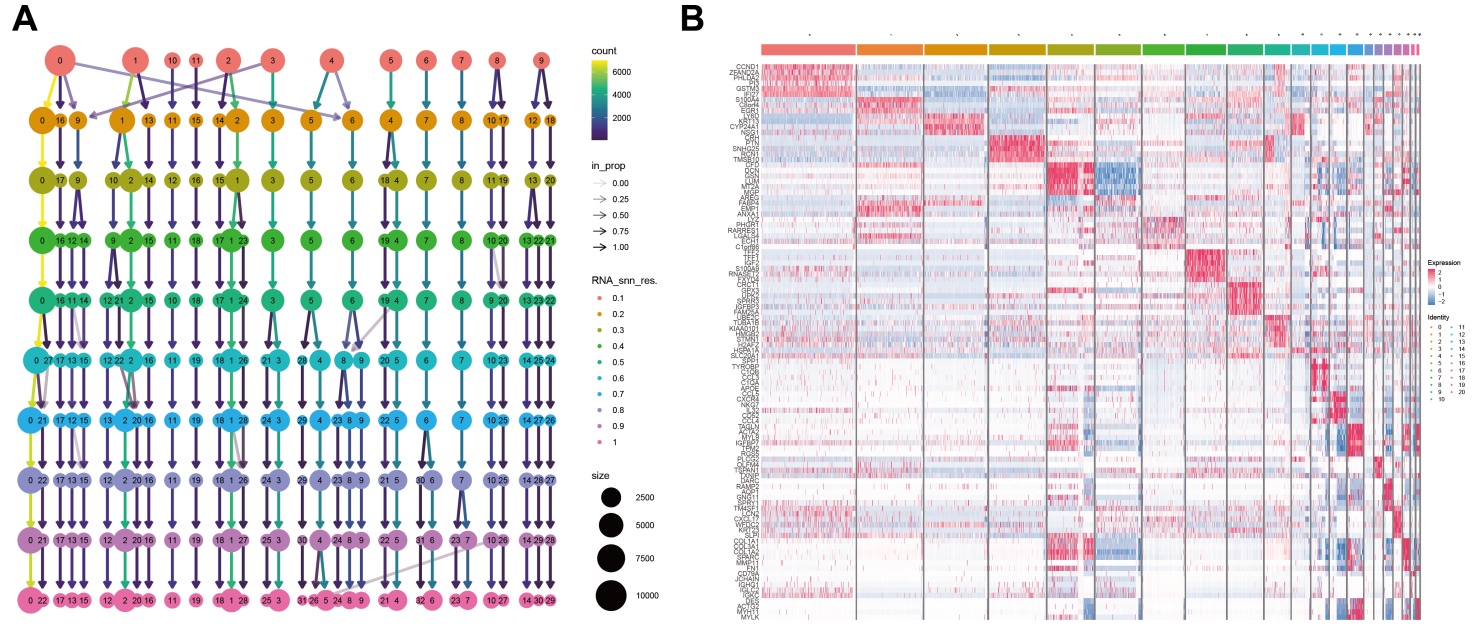
**

**Figure S2. Clustering and DEGs Across Resolutions.**

Note: (A) A cell clustering tree diagram generated using the Clustree package illustrates the evolution of clustering from low to high resolution. Arrows indicate cluster splits or merges, and colors represent the proportion of cells within each cluster; (B) A heatmap displaying the top five DEGs for each of the 21 cell clusters identified at a resolution of 0.3, with colors ranging from red (high expression) to blue (low expression). All analyses presented in this figure are based on single-cell RNA-seq data from GSE135337 (Tumor: GSM4006644, GSM4006645, GSM4006646, GSM4006647, GSM4006648, and GSM4751267; Normal: GSM5329919) and GSE192575 (Sen: GSM5751919; Res: GSM5751918).


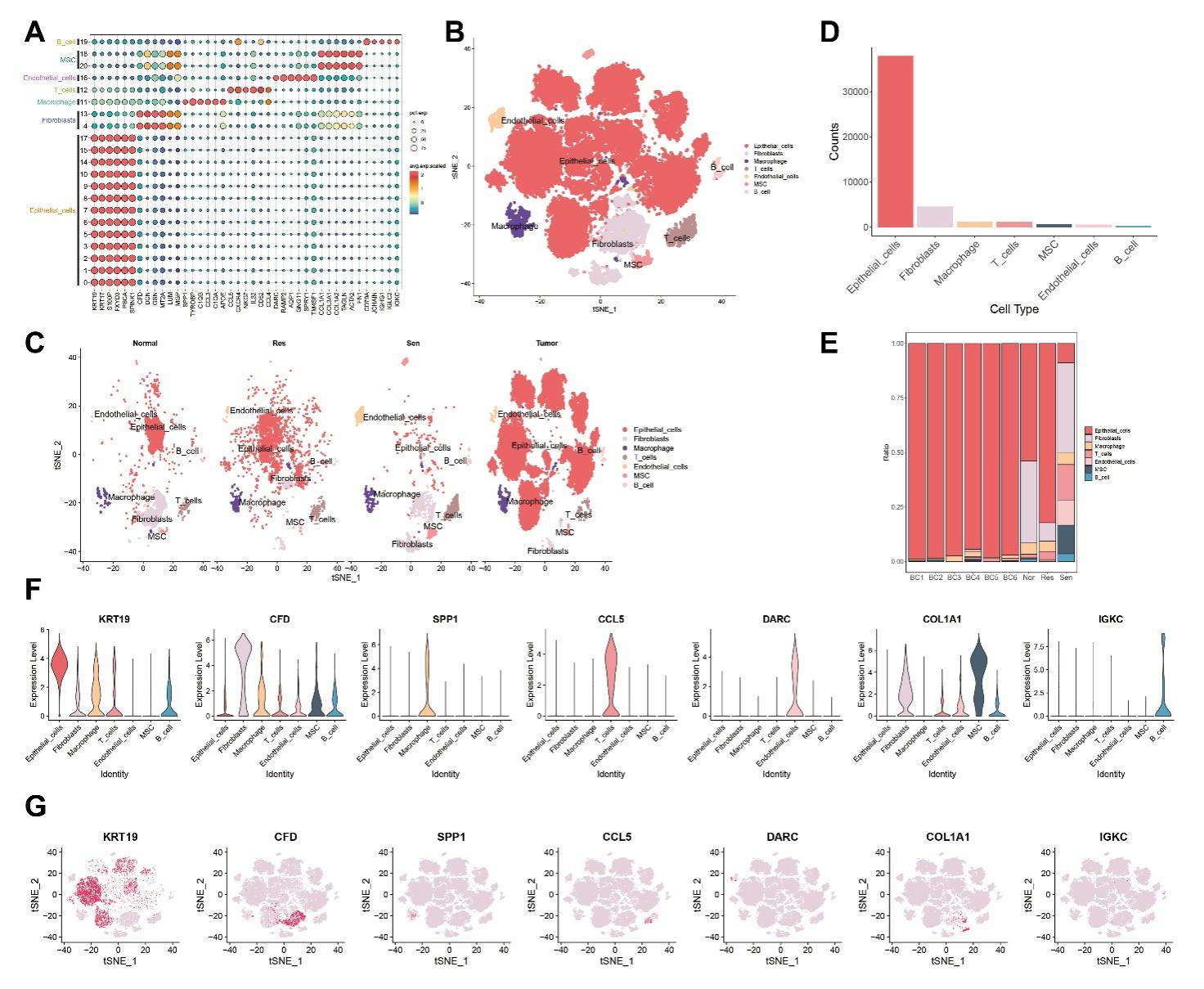


**Figure S3. scRNA-seq Reveals the Distribution and Characteristics of Cell Types in the BC Microenvironment.**

Note: (A) Dot plot showing the expression patterns of marker genes across cell populations; (B) Visualization of cell populations using t-SNE analysis; (C) Distribution of cellular heterogeneity across cisplatin-sensitive (Sen, n = 1), cisplatin-resistant (Res, n = 1), tumor (Tumor, n = 6), and normal (Normal, n = 1) samples; (D) Quantitative analysis of different cell types across samples; (E) Proportional distribution of cell populations among different groups; (F) Boxplot illustrating marker gene expression across cell types; (G) Feature plots showing the expression levels of marker genes in different cell types (KRT19: epithelial cells; CFD: fibroblasts; SPP1: macrophages; CCL5: T cells; DARC: endothelial cells; COL1A1: MSCs; IGKC: B cells). All analyses in this figure are based on single-cell RNA-seq data from GSE135337 (Tumor: GSM4006644, GSM4006645, GSM4006646, GSM4006647, GSM4006648, and GSM4751267; Normal: GSM5329919) and GSE192575 (Sen: GSM5751919; Res: GSM5751918).

**
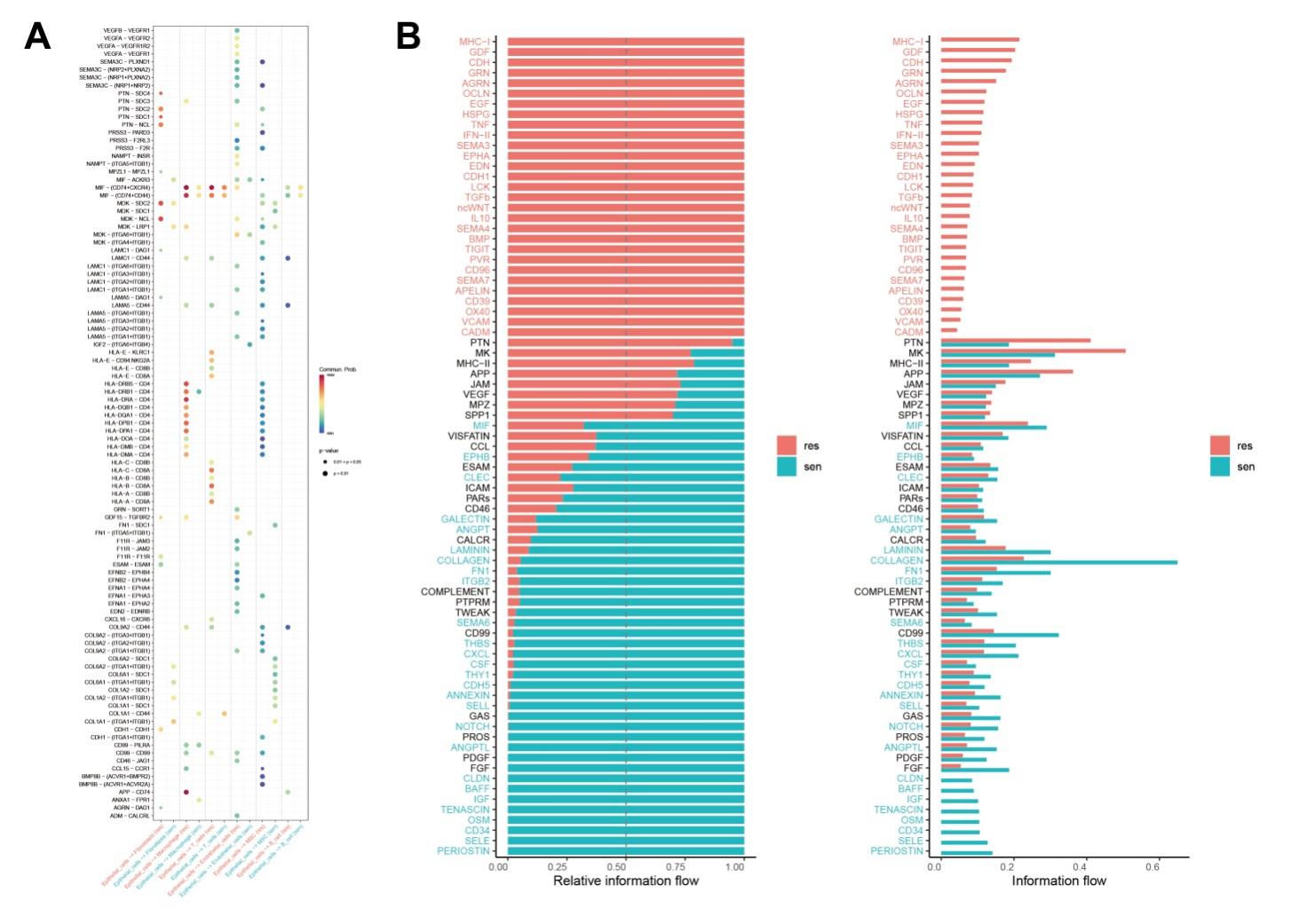
**

**Figure S4. CellPhoneDB Analysis Reveals Receptor-Ligand Interactions Associated with Cisplatin Resistance in BC.**

Note: (A) Receptor-ligand interactions between the Res and Sen groups analyzed using the CellPhoneDB database; (B) Trends in signal pathway information flow across different groups: cisplatin-sensitive (Sen, n=1), cisplatin-resistant (Res, n=1), tumor (Tumor, n=6), and normal (Normal, n=1). All analyses in this figure are based on single-cell RNA-seq data from GSE135337 (Tumor: GSM4006644, GSM4006645, GSM4006646, GSM4006647, GSM4006648, and GSM4751267; Normal: GSM5329919) and GSE192575 (Sen: GSM5751919; Res: GSM5751918).

**
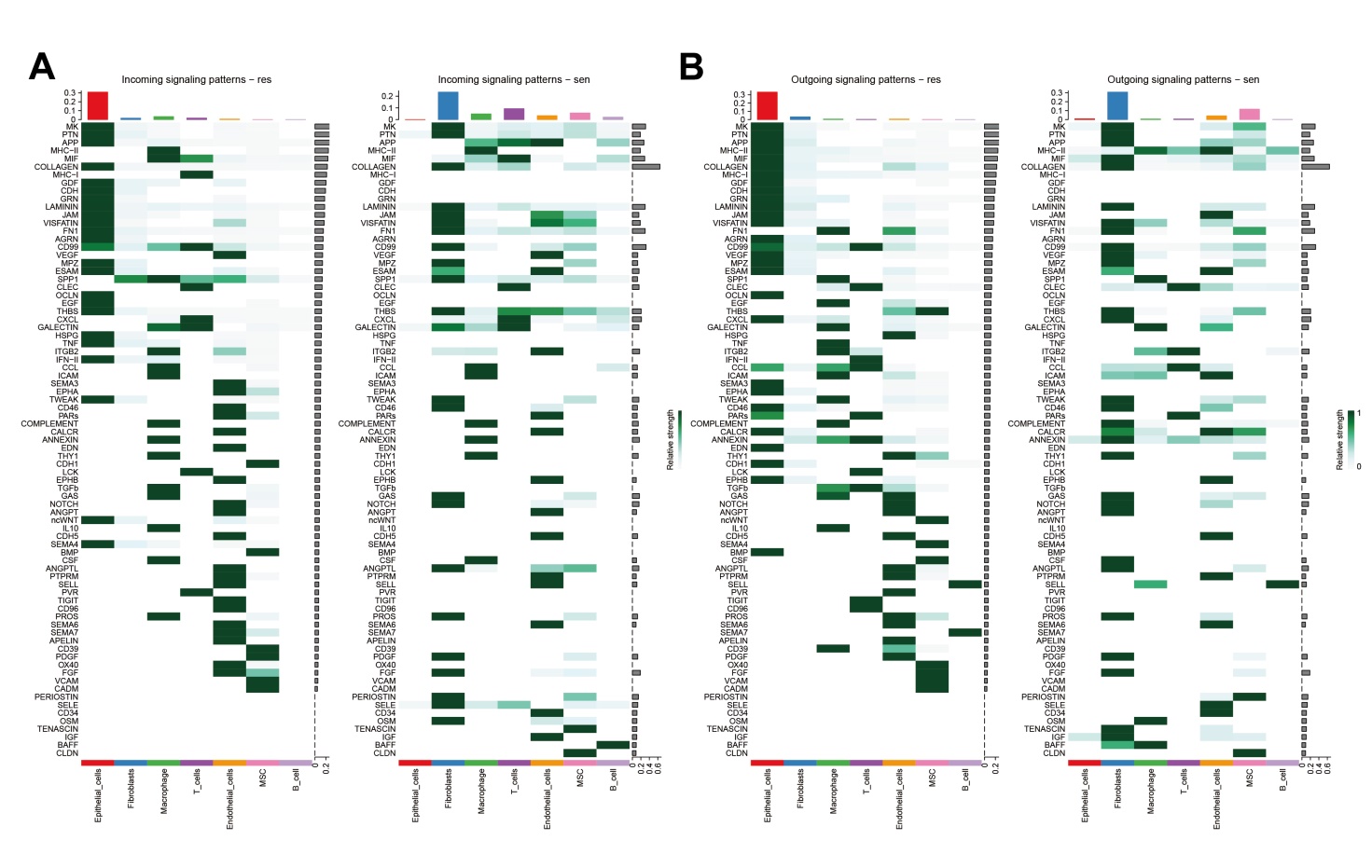
**

**Figure S5. CellChat Network Analysis of Cisplatin-Resistant and Cisplatin-Sensitive Groups in BC.**

Note: (A) Changes in incoming signaling pathways between the resistant group (Res) and the sensitive group (Sen); (B) Changes in outgoing signaling pathways between the resistant group (Res) and the sensitive group (Sen). Cisplatin-sensitive group (Sen, n=1), cisplatin-resistant group (Res, n=1), tumor group (Tumor, n=6), and normal group (Normal, n=1). All analyses in this figure are based on single-cell RNA-seq data from GSE135337 (Tumor: GSM4006644, GSM4006645, GSM4006646, GSM4006647, GSM4006648, and GSM4751267; Normal: GSM5329919) and GSE192575 (Sen: GSM5751919; Res: GSM5751918).

**
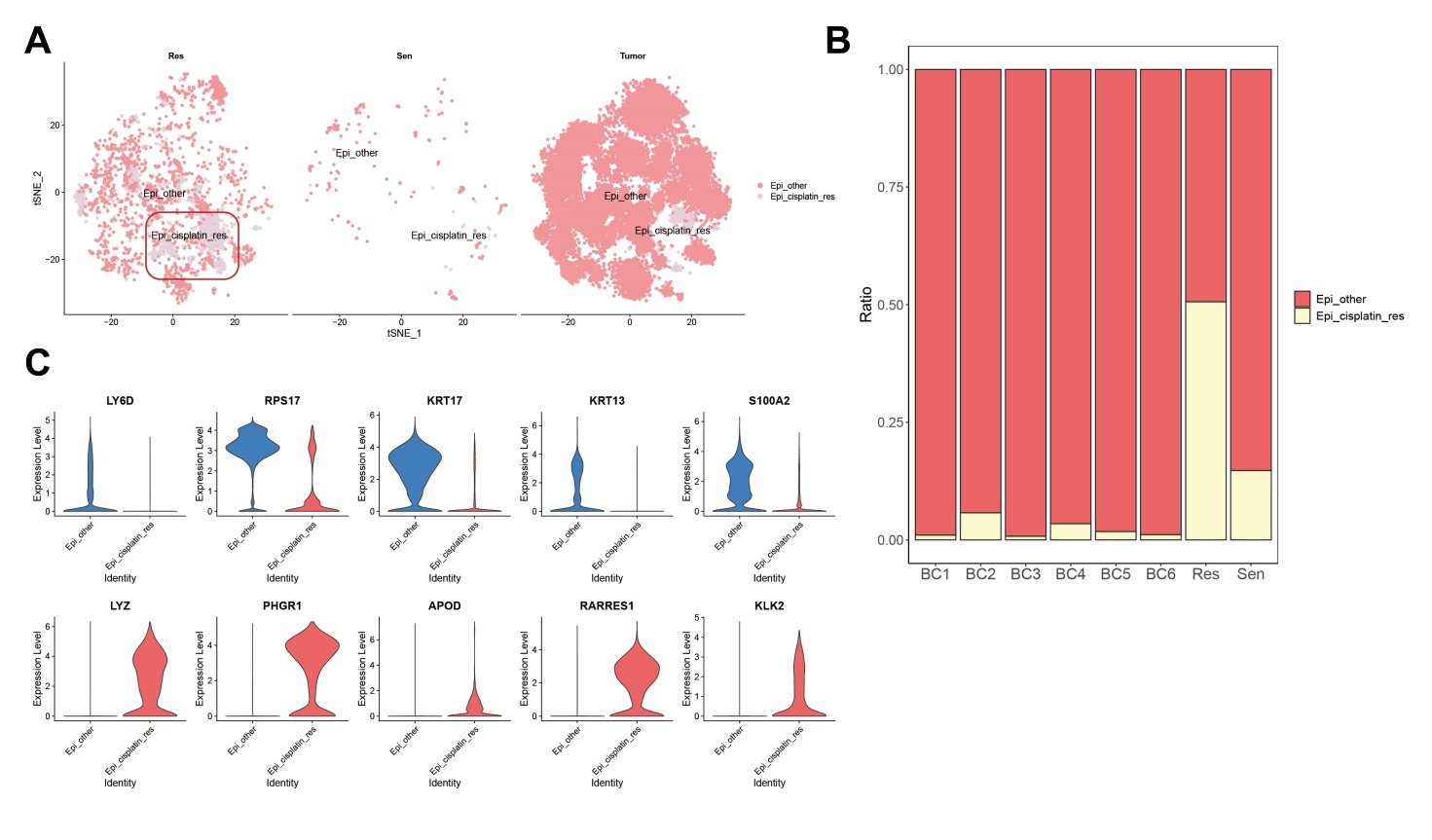
**

**Figure S6. Characterization and Marker Gene Expression Analysis of Epi_cisplatin_res and Epi_other in BC.**

Note: (A) Distribution of Epi_cisplatin_res and Epi_other in the t-SNE dimensionality reduction space; (B) Proportion of Epi_cisplatin_res and Epi_other subpopulations in different samples; (C) Box plots displaying the expression of marker genes across various cell types.


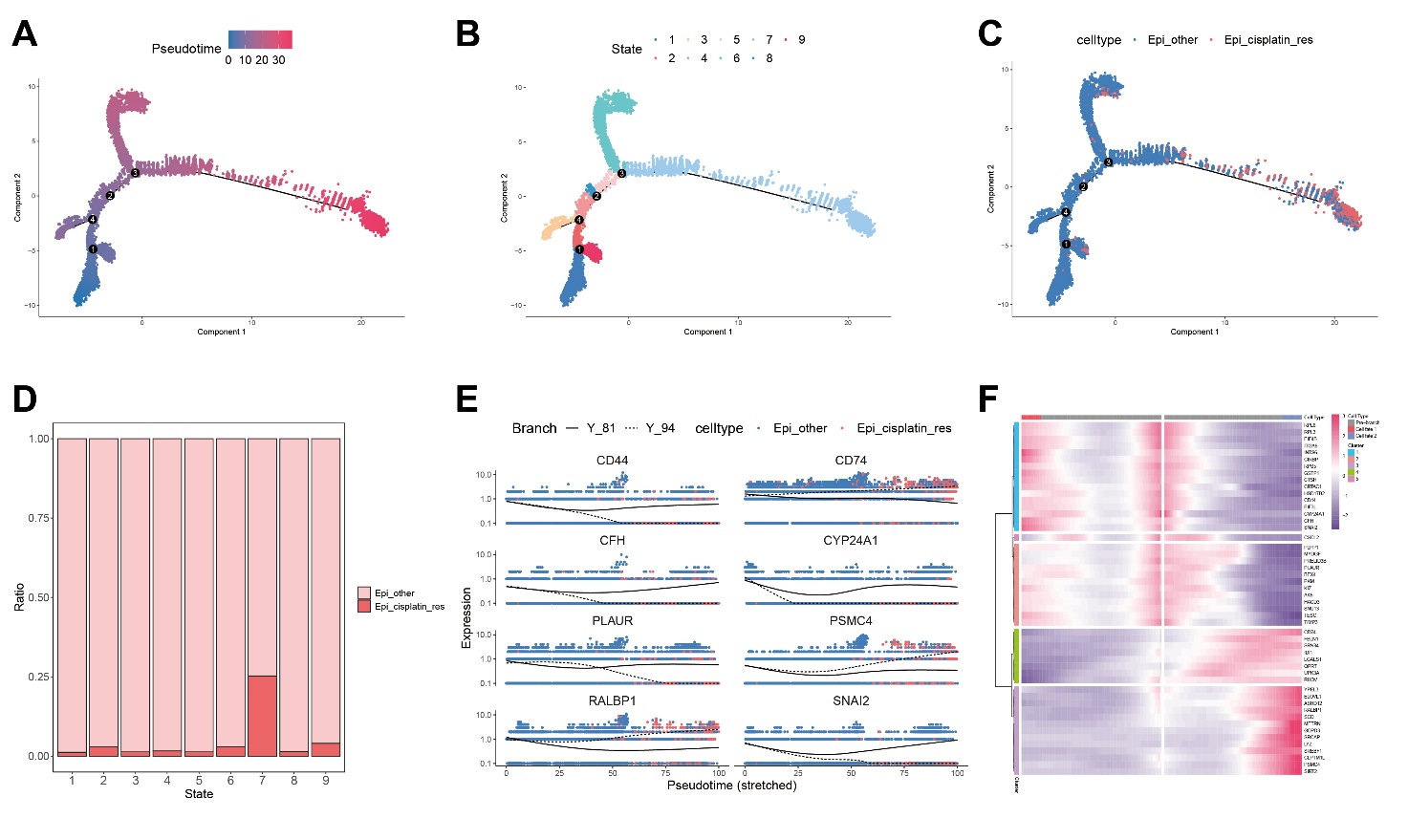


**Figure S7. Pseudotime Analysis of BC Epithelial Cells Reveals Differentiation Trajectories and Dynamic Changes in Transcription Factors.**

Note: (A) Pseudotime trajectory analysis of epithelial cell subpopulations using Monocle 2, illustrating transcriptional state changes; (B) Pseudotime distribution of epithelial cell subpopulations across different states; (C) Distribution of Epi_cisplatin_res and Epi_other subpopulations in the pseudotime analysis; (D) Proportion of Epi_cisplatin_res and Epi_other subpopulations across states; (E) Expression trends of key transcription factors along the pseudotime trajectory; (F) Heatmap showing dynamic expression changes of resistance-related genes during the pseudotime progression.
